# Synthesis, Biological Evaluation, and Molecular Modeling Studies of New Thiadiazole Derivatives as Potent P2X7 Receptor Inhibitors

**DOI:** 10.3389/fchem.2019.00261

**Published:** 2019-04-30

**Authors:** Daniel T. G. Gonzaga, Felipe H. Oliveira, N. L. von Ranke, G. Q. Pinho, Juliana P. Salles, Murilo L. Bello, Carlos R. Rodrigues, Helena C. Castro, Hellen V. C. M. de Souza, Caroline R. C. Reis, Rennan P. P. Leme, João C. M. Mafra, Luiz C. S. Pinheiro, Lucas V. B. Hoelz, Nubia Boechat, Robson X. Faria

**Affiliations:** ^1^Departamento de Síntese de Fármacos Manguinhos, Fundação Oswaldo Cruz, Instituto de Tecnologia em Fármacos, Farmanguinhos-Fiocruz, Rio de Janeiro, Brazil; ^2^Instituto Biomédico, Centro Universitário Estadual da Zona Oeste, Rio de Janeiro, Brazil; ^3^Laboratório de Toxoplasmose e Outras Protozooses, Fundação Oswaldo Cruz, Instituto Oswaldo Cruz, Rio de Janeiro, Brazil; ^4^Departamento de Fármacos e Medicamentos, Faculdade de Farmácia, Universidade Federal do Rio de Janeiro, Rio de Janeiro, Brazil; ^5^Laboratório de Antibióticos, Bioquímica, Ensino e Modelagem Molecular–LABiEMol, Universidade Federal Fluminense, Niterói, Brazil

**Keywords:** P2X7 receptor, thiadiazole, pyrazole, dye uptake, IL-1β release, paw edema, molecular docking

## Abstract

Twenty new 2-(1*H*-pyrazol-1-yl)-1,3,4-thiadiazole analogs were synthetized to develop P2X7 receptor (P2X7R) inhibitors. P2X7R inhibition *in vitro* was evaluated in mouse peritoneal macrophages, HEK-293 cells transfected with hP2X7R (dye uptake assay), and THP-1 cells (IL-1β release assay). The 1-(5-phenyl-1,3,4-thiadiazol-2-yl)-1*H*-pyrazol-5-amine derivatives **9b**, **9c**, and **9f**, and 2-(3,5-dimethyl-1*H*-pyrazol-1-yl)-5-(4-fluorophenyl)-1,3,4-thiadiazole (**11c**) showed inhibitory effects with IC_50_ values ranging from 16 to 122 nM for reduced P2X7R-mediated dye uptake and 20 to 300 nM for IL-1β release. In addition, the *in vitro* ADMET profile of the four most potent derivatives was determined to be in acceptable ranges concerning metabolic stability and cytotoxicity. Molecular docking and molecular dynamics simulation studies of the molecular complexes human P2X7R/**9f** and murine P2X7R/**9f** indicated the putative intermolecular interactions. Compound **9f** showed affinity mainly for the Arg268, Lys377, and Asn266 residues. These results suggest that 2-(1*H*-pyrazol-1-yl)-1,3,4-thiadiazole analogs may be promising novel P2X7R inhibitors with therapeutic potential.

## Introduction

Pyrazole (Küçükgüzel and Senkardeş, 2015; Faria et al., 2017) and the 1,3,4-thiadiazole derivatives show several biological activities (Hu et al., 2014), including anti-viral (Gan et al., 2017), anti-bacterial (Aggarwal et al., 2014), anti-tumoral (Rai et al., 2015), anti-inflammatory (El-Sehemi et al., 2014), anti-cancer (Raj et al., 2015), anti-convulsant (Raj et al., 2017), anti-depressant (Can et al., 2018), and other effects (Pérez-Fernéndez et al., 2014; Shawali, 2014; Ansari et al., 2017; Karrouchi et al., 2018). In addition, the linkage of the pyrazole and 1,3,4-thiadiazole rings, forming the 2-(1*H*-pyrazol-1-yl)-1,3,4-thiadiazole moiety, also affords compounds with many biological activities, such as insecticide (Dai et al., 2016), anti-inflammatory (Bekhit et al., 2008), COX-2 inhibitors (Alegaon et al., 2014), and anti-cancer (Dawood et al., 2013). However, to date, there are no data related to the effect of this 2-(*1H*-pyrazol-1-yl)-1,3,4-thiadiazole group on plasmatic membrane receptors, including the P2X7 receptor (P2X7R), a purinergic receptor.

P2X7R is a ligand-gated ionotropic purinoceptor permeable to Na^+^, K^+^, and Ca^2+^ when activated by extracellular adenosine triphosphate (ATP; North, 2002). Cells from a wide variety of tissues express this receptor (Ralevic and Burnstock, 1998), especially immune cells such as macrophages and microglia (North and Jarvis, 2013). Prolonged exposure to ATP induces the formation of a pore that permeates molecules up to 900 Da. ATP-induced pores associated with P2X7R promote cell death dependent on the carboxyl-terminal domain (North and Jarvis, 2013). Another important activity associated with P2X7R is the maturation and release of interleukin-1β (IL-1β), which is a cytokine related to inflammation and pain signaling (Skaper et al., 2010). Therefore, the biological effects related to P2X7 make this receptor a relevant therapeutic target to develop new anti-inflammatory compounds (Alves et al., 2013; Santana et al., 2015; Bou-Dargham et al., 2017) against rheumatoid arthritis (a chronic inflammatory disorder).

Thus, research institutions and pharmaceutical groups have investigated novel P2X7R inhibitors (Burnstock, 2017; Chen et al., 2018; Young and Górecki, 2018). However, these compounds have failed in clinical assays for rheumatoid arthritis treatment (Stock et al., 2012). This finding supports the search for new compounds exhibiting therapeutic activity against P2X7R (Burnstock, 2017; Chen et al., 2018; Young and Górecki, 2018).

In this study, 20 2-(*1H*-pyrazol-1-yl)-1,3,4-thiadiazole analogs were synthesized (Chapleo et al., 1986; Bastos et al., 2008) to evaluate their inhibitory activity on P2X7R *in vitro* and the acute inflammatory response *in vivo*. The physicochemical properties were also calculated for all compounds. In addition, molecular docking and molecular dynamics (MD) simulations were performed using the most active compound to study the dynamic behavior of P2X7R involved in the inhibition process.

## Biological Assays

### Mammalian Cells

#### Mouse Peritoneal Macrophages

Our protocols adhered to the Ethical Principles in Animal Experimentation adopted by the Brazilian College of Animal Experimentation and approved by the FIOCRUZ Research Ethics Committee (Faria et al., 2005; number L039- 2016). Male mouse (Swiss Webster) peritoneal macrophages were collected from peritoneal cavity lavage and plated for 24 h before dye uptake assays, whole-cell patch clamp experiments, and IL-1β release assays.

#### HEK-293 Cells Transfected With P2X7R

HEK-293 cells expressing human P2X7R were maintained in Dulbecco's modified Eagle's medium (DMEM; Sigma-Aldrich) supplemented with 10% fetal bovine serum (FBS; Prolab, Br) and anti-biotics (50 U/ml penicillin and 50 mg/ml streptomycin) in a humidified 5% CO_2_ atmosphere at 37°C (Faria et al., 2018). A total of 5 × 10^5^ cells were plated at 37°C in a humidified 5% CO_2_ atmosphere for 24 h in a 96-well plate for dye uptake or IL-1β release assays.

### LDH Release Assay

Mouse peritoneal macrophages (5 × 10^5^ cells) and HEK-293 cells transfected with P2X7R (5 × 10^5^ cells) were plated in a 96-well-plate for 24 h before the treatment. Mammalian cells were treated with thiadiazole analogs for 24 h with concentrations ranging from 1 nM to 0.5 mM. The supernatant collected in this assay was used to measure the presence of LDH in the media using a cytotoxicity detection kit (Sigma kit for LDH) according to the manufacturer's instructions (Faria et al., 2018).

### Dye Uptake Assay

HEK-293 cells transfected with hP2X7R and mouse peritoneal macrophages were plated at 2.5 × 10^6^ or 5 × 10^5^ cells/ml, respectively, in 96-well culture plates containing DMEM with 10% FBS and anti-biotics (50 U/ml penicillin and 50 mg/ml streptomycin) in a humidified 5% CO_2_ atmosphere at 37°C for 24 h (Faria et al., 2018).

P2X7R antagonist and thiadiazole analogs (1 nM to 0.5 mM) were incubated for 5 min before ATP (5 mM) treatment for 20 min. In the last 5 min of ATP incubation, propidium iodide for macrophages (PI; 750 nM) or ethidium bromide (EB; 25 μM) for HEK-293 cells was added to all wells. PI dye was excited at a wavelength of 530 nm, and its fluorescence emission was read at a wavelength of 590 nm using an M5 plate reader (Molecular Devices). EB dye was excited at a wavelength of 530 nm and emitted at a wavelength of 620 nm.

### Electrophysiological Measurements

The whole-cell configuration was used in peritoneal macrophages as previously described (Faria et al., 2005). All the experiments used bath and pipette solutions with a series resistance set as 6–11 MΩ. Ionic currents with amplitudes < 1,500 pA were not compensated; however, ionic currents above this level were compensated by 88%. The cell capacitance for peritoneal macrophages was (19 ± 1 pF; *n* = 86), and the recordings were obtained at a holding potential of −60 mV at 37°C.

#### Saline Solutions for Electrophysiology

The saline composition was as follows (in mM): 150 NaCl, 5 KCl, 1 MgCl_2_, 1 CaCl_2_, and 10 HEPES (pH 7.4) for the bath solution and 150 KCl, 5 NaCl, 1 MgCl_2_, 10 HEPES, and 0.1 EGTA (pH 7.4) for the pipette solution (Faria et al., 2018).

#### Drug Application

Ionic currents were studied by applying 1 mM ATP (for 300 s) in the presence or absence of compound **9f** or P2X7R antagonists. A perfusion chamber (RC-24 chamber, Warner Instrument Corp) operating at a rate of 1 ml/min was used in all experiments (Faria et al., 2018).

### Measurements of Intracellular Ca^2+^ Levels

Mouse peritoneal macrophages, PC12 cells, J774 cells, and HEPG2 cells were analyzed by fluorescence microscopy to measure the intracellular Ca^2+^ concentrations ([Ca^2+^]i). Cells were incubated with 2 μM Fura-2-AM (Molecular Probes) for 30 min, and the [Ca^2+^]_i_ mobilization was measured in the F340/F380 ratios with a FlexStation 3 multimode microplate reader (Molecular Devices). Cells were plated in translucent 96-well plates (BD Falcon) for 15 min and then washed and incubated in a saline solution with 150 NaCl, 5 KCl, 1 MgCl_2_, 1 CaCl_2_, and 10 HEPES (pH 7.4) for 30 min before measurements of [Ca^2+^]i. The Ca^2+^ influx was induced by stimulating cells with P2 receptor agonists. P2 receptor antagonists were added 10 min before P2 receptor agonist addition. Ca^2+^ mobilization was measured as the area under the curve (AUC) after ionomycin (1 μM) or P2 receptor agonist stimulation. Ionomycin was considered a positive control, and the other recordings were normalized in relation to the AUC.

### IL-1β Enzyme-Linked Immunosorbent Assay

P2X7R-mediated IL-1β release was obtained from differentiated THP-1 cells stimulated with lipopolysaccharide (LPS) before ATP addition. These cells were plated at 2 × 10^5^ cells/well in 96-well culture plates maintained in RPMI supplemented with 10% FBS, penicillin (100 U/ml), and streptomycin (100 mg/ml) in a humidified 5% CO_2_ atmosphere at 37°C. THP-1 cells were differentiated with 500 ng/ml phorbol 12-myristate 13-acetate (PMA) and 10 ng/ml IFN-γ cotreatment for 24 h. These cells were activated with 25 ng/ml LPS for 4 h. The second stimulation with ATP (5 mM) occurred in the last 30 min of the LPS incubation (Faria et al., 2018). P2X7R antagonists (BBG and A740003) and thiadiazole analogs were added 30 min before the ATP stimulus. The supernatants were collected, centrifuged (1,000 rpm for 5 min at 4°C), and stored at −70°C after LPS incubation. IL-1β was quantified using a standard kit (ABCAM, Cambridge).

### Caco-2 Cell Culture and Treatments

Corning® Costar® Transwell plates (Sigma-Aldrich, St. Louis, MO, USA) were used for seeding Caco-2 cells at 3 × 10^5^ cells/well bathed with DMEM supplemented with 10% FSB according to Faria and collaborator in 2018 (Faria et al., 2018). This culture was maintained for up to 21 days in a humidified incubator at 37°C and 5% CO_2_. Thiadiazole analog, vinblastine (poor permeability control), and propranolol (high permeability control) stock solutions, all at a concentration of 100 mM, were prepared in Hanks' balanced salt solution (HBSS) containing 25 mM HEPES at pH 7.4 with 0.5% (v/v) DMSO. Transport buffer (0.3 ml) was added to each well to equilibrate the cells with the transport buffer. A 24-well enhanced recovery plate containing 1 ml of transport buffer (pH 7.4) was substituted for the feeder tray. The transport buffer in the apical wells was removed, and 0.3 ml of a solution containing thiadiazoles **9b**, **9c**, **9f**, or **11c**; vinblastine; or propranolol was added. Then, the cells were replaced for incubation for 60 min. Lucifer yellow concentrations in the donor and acceptor wells were measured in the last of this incubation. Lucifer yellow was measured using an M5 plate reader (Molecular Probes) at an excitation wavelength of 485 nm and an emission wavelength of 530 nm.

### pH-Dependent Solubility of 9f Analog

To measure the kinetic solubility of analog **9f**, DMSO stock solutions (5 μl, in triplicate) with concentrations from 1 to 250 μM were added to 995 μl of buffer (pH 2.0 hydrochloride, 4.0–100 mM citrate buffer, and 7.4–100 mM phosphate buffer) in a 96-well plate for 2 h at room temperature. DMSO stock solutions (5 μl) were added into a 995-μl acetonitrile/buffer (1:1) mixture to prepare the calibration standard solutions. The reaction samples were centrifuged (10,000 rpm, 10 min, 25°C) and diluted 1:1 with acetonitrile (Faria et al., 2018).

### Distribution Coefficient (Log D) in Octanol/PBS pH 7.4

Octanol and Phosphate-buffered saline (PBS) solution at a ratio of 1:1 (v/v) at pH 7.4 was shaken mechanically for 24 h to reach presaturation. Crescent octanol volumes (100–400 μl) were added to PBS (396 μl) and thiadiazole **9f** (25 mM) in a volume of 4 μl. This solution was shaken for 2 h and centrifuged (3,000 rpm) for 5 min, and the PBS layer was collected 1 h later. To measure the PBS layer absorbance, a 100-μl aliquot (396 μl of PBS containing 4 μl of **9f** + 400 μl of acetonitrile) was partitioned with acetonitrile (100 μl; Faria et al., 2018).

### *In vitro* Stability Assays in Liver Microsomes

Thiadiazole **9f** stability was evaluated in liver microsomes from male mice and humans according to Faria et al. in 2018 (Faria et al., 2018). Both solutions contained a final protein concentration of 0.5 mg/ml (0.1 M phosphate buffer) at pH 7.4. Microsomes were preincubated with **9f** (1 μM) and DMSO (0.5 μM) at 37°C before NADPH (1 mM) addition. A control for the reaction was a buffer containing 0.1 M phosphate at pH 7.4 in a final volume of 50 μl. Diazepam and verapamil were used as positive controls for mice and humans, respectively. Both types of microsomes were treated with **9f** for 0, 5, 15, 30, and 45 min and the negative control [minus Nicotinamide adenine dinucleotide phosphate (NADPH)] for 45 min. To stop the reactions, methanol (50 μl) was added at the appropriate time points, and the samples were centrifuged (1,640 × *g*) for 20 min at 4°C to prevent protein precipitation.

*In vitro* intrinsic clearance (CLint mic) for the metabolism of **9f** in mouse and human liver microsomes was calculated using the equations below:

Half-life (t1/2) (minutes) = 0.693/k (1)

V(μL/mg) = volume of the incubation solution (μl)/protein in the incubation solution (mg) (2)

Intrinsic clearance (CLint) (μl/minutes/mg protein) = V × 0.693/t1/2 (3) according to the manufacturer's instructions.

### *In vivo* Experimental Assays With Mice

Our protocols using Swiss Webster mice 4–5 weeks old adhered to the Ethical Principles in Animal Experimentation adopted by the Brazilian College of Animal Experimentation and approved by the FIOCRUZ Research Ethics Committee (Faria et al., 2018) (number LW-5814).

#### Carrageenan- and ATP-Induced Paw Edema

Mouse paw edema was stimulated with carrageenan (300 μg/paw) saline suspension for 60 min or ATP (10 mg/paw) for 30 min. Treatment with diclofenac (100 μg/kg), oxidized ATP (100 μg/kg), and analog **9f** (0.001–1 mg/kg) was intraperitoneally administered 60 min prior to the intrathecal administration of carrageenan or ATP. A plethysmometer (UGO Basil, Italy) was used to measure the edema before inductor injection, and the relative paw edema increased after carrageenan or ATP action.

### Statistical Analyses

PRISM® software was used for analyzing all data (GraphPad Inc., San Diego, CA, USA). The results were expressed as the mean ± standard deviation of the mean (SDM) measured in triplicate and executed on at least three independent days. The D'Agostino and Pearson normality tests were used to estimate whether the samples followed a Gaussian distribution. When the data followed a Gaussian distribution, analysis of variance (ANOVA) was applied consecutively to Tukey's test. Otherwise, the non-parametric Kruskal–Wallis test was applied consecutively to Dunn's test. All tests were two-tailed and are specified in the figure legends. *P*-values < 0.05 were considered statistically significant.

## In silico

### ADMET Properties

Some physicochemical, pharmacokinetic, and toxicological parameters of the molecules were calculated using the programs Osiris® (Actelion Pharmaceuticals Ltda; http://www.organic-chemistry.org/prog/peo/) and ADMET Predictor® (Simulation Plus) since these programs use different techniques and provide additional results.

#### Comparative Modeling

The amino acid sequences of the human (hP2X7R; UniProtKB ID: Q99572) and murine (mP2X7R; UniProtKB ID: Q9Z1M0) P2X7R were obtained from the EXPASY proteomic server (http://ca.expasy.org/). The subunit sequences of each receptor were submitted to the LOMETS server (Wu and Zhang, 2007), which constructs 3D models by collecting high-scoring structural templates from 11 threading programs (CEthreader, FFAS3D, HHpred, HHsearch, MUSTER, PRC, PROSPECT2, PPAS, SP3, SparksX, and wMUSTER). Subsequently, the construction of the P2X7R models was performed by Modeller 9.18 software (Sali and Blundell, 1993) that employs spatial restriction techniques based on the 3D template structure. These preliminary models, hP2X7R and mP2X7R, were refined in the same software using seven and five cycles of the default optimization protocol, respectively. The structural evaluation of the models was carried out using Procheck software (stereochemical quality analysis) (Laskowski et al., 1993).

### Ligand Preparation

Chemicalize® software (ChemAxon; Swain, 2012) was used to determine the most favorable non-ionized state of compound **9f** at physiological pH 7.4. The molecular model of compound **9f** was built using the program Spartan10 v.1.0.1 (Wavefunction Inc., Irvine, CA, USA, 2000) followed by a conformer distribution to find the local energy minimum conformers using the MMFF94 force field (Halgren, 1996). Thus, a low-energy conformer was selected to calculate the equilibrium geometry using the semiempirical method RM1 (Recife Model 1; Rocha et al., 2006).

### Molecular Docking

The human and murine P2X7 models were employed in molecular docking using the algorithm MolDock (Thomsen and Christensen, 2006) with the Molegro Virtual Docker (MVD) 6.0 program (CLC Bio, 8200, Aarhus, Denmark). It was applied with a grid resolution of 0.30 Å, and the score function was the MolDock score (GRID) algorithm. The partial charges were assigned as reported by the MVD charges scheme. The search algorithm used was the MolDock Optimizer with a search space around the area of the ATP binding site and the allosteric site juxtaposed with the ATP binding pocket located in the P2X7R pore. The ligand evaluation was considered in relation to internal electrostatic interactions (ESs), internal hydrogen bonds (H-bonds), and Sp2–Sp2 torsions. Every molecular docking procedure was performed with 50 runs and the same parameter set (population size = 50, max iterations = 2,000, scaling factor = 0.50, and crossover rate = 0.90) followed by energy minimization. The best binding poses were selected for the following molecular modeling steps.

### Lipid Membrane Model

The P2X7 protein membrane systems were prepared by the CHARMM-GUI Membrane Builder (Jo et al., 2014). Therefore, the protein was previously oriented in the membrane by using the OMP server (Lomize et al., 2012), which calculates the rotational and translational positions of the P2X7 protein in the layer. Each protein simulation system was built by the Membrane Builder insertion method, which creates a hole to insert a protein in a lipid bilayer model. This approach is useful for proteins such as the P2X7R, which presents cylindrical and symmetrical shapes (Jo et al., 2014). The membrane–protein systems were then solvated in a rectangular water box of 170 × 100 × 100 Å^3^ dimensions. A pore of water was generated in the protein P2X7 model since it is an ion channel and, thus, able to accommodate water molecules inside. Sufficient Na^+^ anions were added to the system to achieve charge neutrality by the ion-accessible volume and the total charge of the system. The composition of the membrane bilayer lipid model was prepared based on information from the work by van Meer et al. (2008). The lipid composition is presented in Supplemental Table 1.

### Setup and Molecular Dynamics Simulations

Three-dimensional models of the human and mouse P2X7Rs (hP2X7R and mP2X7R) with lipid membrane and water molecules, both in apo and in complex with the inhibitor, were used to study the MD of the free (hP2X7R_APO_ and mP2X7R_APO_) and bound (hP2X7R_9f_ and mP2X7R_9f_) aqueous systems. Subsequently, the ionization states of the amino acids at pH 7.0 in aqueous medium were determined. The Resp Esp charge Derive (R.E.D.) server (Vanquelef et al., 2011) was used to calculate the partial charges of the ligand, and its topology was prepared by applying the ACPYPE program. The GROMACS 5.1.4 package (Abraham et al., 2015) was applied in the MD simulations using the CHARMM36 force field (Best et al., 2012).

The MD simulations were performed under periodic boundary conditions and in the NpT ensemble. The Nosé–Hoover thermostat was used to maintain the temperature at 310 K (Nosé and Klein, 1983), and the Parrinello–Rahman barostat was applied to isotropic pressure (Parrinello and Rahman, 1980, 1981). The particle mesh Ewald method (Darden et al., 1993) was applied to electrostatic interactions with a cutoff of 1.4 nm. The Lennard–Jones interaction terms (Berendsen et al., 1995) were also calculated with a cutoff at 1.4 nm. Constraints on all bonds and water molecules were performed with the LINCS (Hess et al., 1997) and SETTLE (Miyamoto and Kollman, 1992) algorithms, respectively. The integration time step was performed every 2.0 fs. The molecular system equilibrations during 2.0 ns were carried out to relax the water molecules around the position-restrained **9f**–P2X7R molecular complex. Finally, without position restraints, the MD simulation production run was performed over 200 ns.

### Analyses of the P2X7R Structures and Dynamics in the Free and Bound Systems

The MD simulation results were analyzed for the molecular systems hP2X7_APO_, hP2X7_9f_, mP2X7R_APO_, and mP2X7R_9f_. The secondary structure (SS) elements were calculated by the program DO_DSSP, root mean square deviations (RMSDs) were calculated using the program G_RMS, root mean square fluctuations (RMSFs) were calculated by the program G_RMSF, and radius of gyration (Rg) was determined using the program G_GYRATE. All these programs are present in the GROMACS 5.1.4 package (Abraham et al., 2015). The SS elements, RMSDs, and Rg were estimated for the entire 200 ns of MD simulation time, while the RMSF was calculated for the last 150 ns of the MD simulation time. The graphs were plotted using the program XMGRACE 5.1.19 (Turner, 2005).

### Intermolecular Hydrogen Bonds Analysis in the P2X7R-Bound Systems

During the last 150 ns of the MD simulation, the intermolecular hydrogen bond (H-bond) interactions of compound **9f** with the amino acids of the hP2X7 and mP2X7 receptors were analyzed. This calculation was performed using the G_HBOND program of the GROMACS 5.1.4 package (Abraham et al., 2015).

### Analysis of the Correlated Movements in the Free and Bound Receptor Systems

The correlated movements of the amino acid residues in the hP2X7R_APO_, hP2X7R_9f_, mP2X7R_APO_, and mP2X7R_9f_ molecular systems were performed using the generalized cross-correlation. The calculation was applied to the atomic coordinates of the Cα-atoms (backbone) during the last 150 ns of the MD simulation. This method was applied using the approach developed by the Grubmüller group and submitted to the G_CORRELATION program (Lange and Grubmüller, 2006) available in the GROMACS 3.3 package (Lindahl et al., 2001).

### Principal Component Analysis of the Receptor Motion in the Free and Bound Systems

The program G_COVAR of the GROMACS 5.1.4 (Abraham et al., 2015) package was applied to calculate the principal components of the P2X7R motions by covariance matrix calculus and its diagonalization during the last 150 ns of the MD simulation. The Cα-atoms were used to calculate the MD simulation trajectories. The prediction was performed using the program G_ANAEIG of the GROMACS package 5.1.4 (Abraham et al., 2015). The MD simulation trajectories were aligned based on the Cα-atoms, avoiding the rotation and translation of the receptor structure. Thus, the MODVECTOR.py script was used to generate the porcupine plot derived from the first principal component calculated by the G_ANAEIG module. The PYMOL 1.5.0.3 software was used to make the images of the porcupine projections (DeLano, 2002; Delano and Bromberg, 2004; Karasawa and Kawate, 2016).

## Results

### Synthesis

**Scheme 1:**
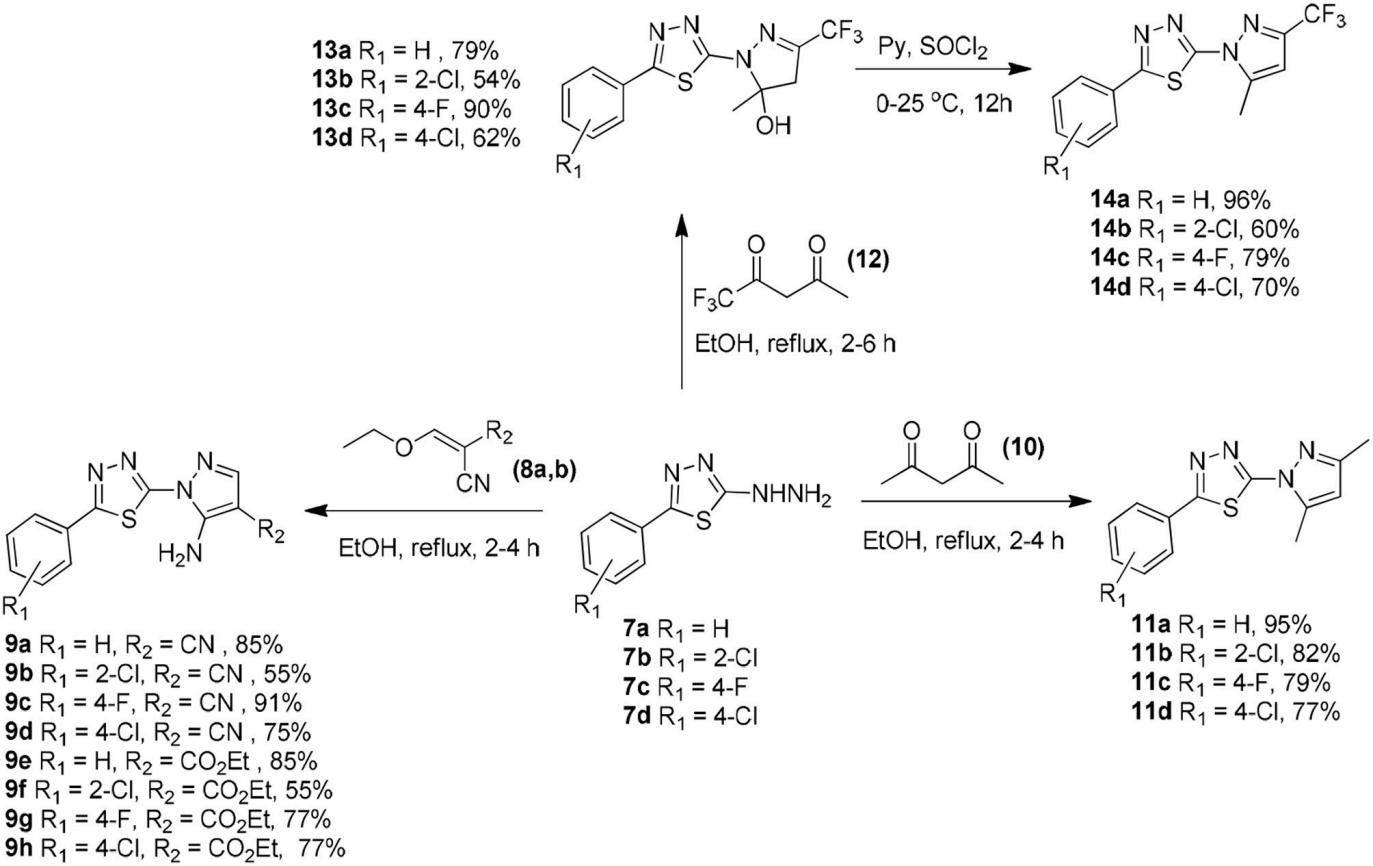
Synthetic routes to obtain 2-(1*H*-pyrazol-1-yl)-1,3,4-thiadiazole derivatives **7a–d**, **9a–h**, **11a–d**, **13a–d**, and **14a–d**.

The chemistry of the analogs is summarized in the Supplemental Materials and Methods—**Chemistry section**. The synthetic routes for preparing compounds **7a–d**, **9a–h**, **11a–d**, **13a–d**, and **14a–d** are shown in Scheme 1. The 2-hydrazinyl-5-phenyl-1,3,4-thiadiazole intermediates (**7a-d**) could easily be prepared in good yields as described in the literature (Chapleo et al., 1986).

The 5-amino-1-(5-phenyl-1,3,4-thiadiazol-2-yl)-1*H*-pyrazole-4-carbonitrile **(9a–d)** or the ethyl 5-amino-1-(5-phenyl-1,3,4-thiadiazol-2-yl)-1*H*-pyrazole-4-carboxylate **(9e–h)** compounds were prepared in 55–91% yield *via* the Michael addition of **7a–d** (2.0 mmol) to 2-(ethoxymethylene)malononitrile **(8a)** or ethyl (ethoxymethylene)cyanoacetate **(8b)** (2.4 mmol), respectively, in ethanol under reflux for 2–4 h (Silva et al., 2016).

The 2-(3,5-dimethyl-1*H*-pyrazol-1-yl)-5-phenyl-1,3,4-thiadiazole compounds **(11a–d)** were prepared from **7a–d** (2.0 mmol) through a reaction with 2,4-pentanedione **(10)** (2.4 mmol) in 77–95% yield in ethanol under reflux for 2–4 h (Aydin et al., 2014). The reaction of **7a–d** (2.0 mmol) with 1,1,1-trifluoropentane-2,4-dione in ethanol under reflux for 2–4 h afforded the 1,3,4-thiadiazol-2-yl-4,5-dihydro-1*H*-pyrazol-5-ol compounds **(13a–d)**. Treatment of **13a–d** with thionyl chloride for 12 h afforded the respective pyrazole derivatives **(14a–d)** in 60–96% yield (Bastos et al., 2008).

The obtained compounds were characterized using ^1^H nuclear magnetic resonance (^1^H NMR), ^13^C NMR, ^19^F NMR, infrared spectroscopy (FTIR), and high-resolution mass spectrometry (HRMS).

### Biological Assays

Twenty thiadiazole derivatives were analyzed (10 μM) concerning their antagonistic effect against P2X7R dye uptake. Analogs **9b**, **9c**, **9f**, and **11c** exhibited inhibition percentages comparable to that of Brilliant Blue G (BBG; Table 1).

**Table 1:** Inhibitory effects of thiadiazole derivatives on P2X7R activity.

**Compound**	**% Inhibition (a)**	**% LDH release (b)**
9a	89.2 ± 2.2	58.1 ± 4.8
9b	30 ± 0.6	15.8 ± 1.3
9c	28.25 ± 0.7	10 ± 0.7
9d	62.25 ± 4.02	46.3 ± 5.12
9e	90.2 ± 0.4	36.3 ± 11.0
9f	15.7 ± 0.6	42.5 ± 0.4
9g	90.75 ± 1.1	22.6 ± 0.3
9h	64 ± 3.7	15 ± 1.9
11a	92.5 ± 0.3	34.1 ± 2
11b	89.7 ± 2.6	45.7 ± 1.7
11c	29.2 ± 0.3	24 ± 1.7
11d	69.75 ± 0.7	36.94 ± 10.6
13a	90.5 ± 1.4	8 ± 0.6
13b	90.7 ± 1.6	19.6 ± 0.6
13c	92 ± 0.4	45.3 ± 1.4
13d	71 ± 0.2	38.5 ± 6.2
14a	91 ± 1	32 ± 1
14b	92 ± 2	26.6 ± 1.2
14c	92.5 ± 0.4	10.6 ± 0.5
14d	54.7 ± 0.5	43 ± 5
BBG (c)	78.8 ± 3.8	13.2 ± 2.6

*(a) % Inhibition values at 10 μM are expressed as percentage relative to the maximum uptake of propidium stimulated by 1 mM ATP only. Data values are expressed as mean ± SD. All experiments were repeated at least three and five times*.

*(b) % LDH release values at 10 μM are expressed as percentage relative to the maximum LDH release caused by 0.05% Triton X-100 only. Data values are expressed as mean ± SD. All experiments were repeated at least three and four times*.

*(c) BBG concentration of 750 nM*.

*LDH, lactate dehydrogenase; ATP, adenosine triphosphate; BBG, Brilliant Blue G*.

All other analogs did not inhibit ATP-induced dye uptake, inhibited with an antagonistic response inferior to that of BBG, or showed lactate dehydrogenase (LDH) release greater than 20% (Table 1). Thus, the selected thiadiazoles **9b**, **9c**, **9f**, and **11c** exhibited EC_50_ (dye uptake assay) values less than those of the antagonists BBG and A740003 in HEK-293-transfected cells (Table 2). Analog **9f** showed potency eight times greater than that of A740003 and more than 50 times greater than that of BBG. In mouse peritoneal macrophages, only the **9f** analog maintained its high inhibitory potency against P2X7R with an IC_50_ value less than that of A740003.

**Table 2:** Inhibitory effect of thiadiazole derivatives in HEK-293 cells transfected with human P2X7R and mice peritoneal macrophages.

**Compound**	HEK-293 IC_**50**_ (μM)^*^	Macrophages IC_**50**_ (μM)^**^
BBG	0.589 ± 0.012	1.2 ± 0.1
A740003	0.097 ± 0.018	0.108 ± 0.016
9b	0.041 ± 0.008	0.111 ± 0.009
9c	0.056 ± 0.002	0.109 ± 0.011
9f	0.011 ± 0.001	0.016 ± 0.002
11c	0.082 ± 0.005	0.122 ± 0.013

* *IC_50_ values were obtained from concentration–response curves for the ethidium uptake assay*.

** *IC_50_ values were obtained from concentration–response curves for the propidium uptake assay. Data values are expressed as mean ± SD. All experiments were repeated at least three times*.

Thiadiazole analog toxicity was measured for 24 h, providing CC_50_ values of 2,267, 6,076, 3,904, and 3,237 μM for **9b**, **9c**, **9f**, and **11c**, respectively, on peritoneal macrophages. Compared to BBG and A740003, all derivatives caused low toxicity (Table 3).

**Table 3:** Toxic effect of thiadiazole derivatives in mouse peritoneal macrophages.

**Compound**	**Macrophages IC_**50**_ (μM)**
BBG	87 ± 1
A740003	325 ± 6
9b	2267 ± 86
9c	3904 ± 77
9f	6076 ± 102
11c	3237 ± 201

The four synthesized compounds, **9b**, **9c**, **9f**, and **11c**, displayed IL-1β inhibitory activities with IC_50_ values ranging from 20 to 300 nM (Table 4). Analogs **9b**, **9c**, and **11c** were approximately 10 times more potent than BBG. Regarding the A740003 antagonist, **9b**, **9c**, and **11c** exhibited similar potency antagonist. In contrast, analog **9f** demonstrated potency more than 20 and 10 times higher than that of BBG and A740003, respectively (Table 4).

**Table 4:** Antagonistic effects of thiadiazole derivatives against ATP-induced IL-1β release in LPS/IFNc-differentiated human THP-1 cells.

**Compound**	**THP-1 cells IC_**50**_ (μM)**
BBG	1.9 ± 0.3
A740003	0.223 ± 0.018
9b	0.298 ± 0.008
9c	0.279 ± 0.035
9f	0.019 ± 0.003
11c	0.240 ± 0.017

**Figure 1:**
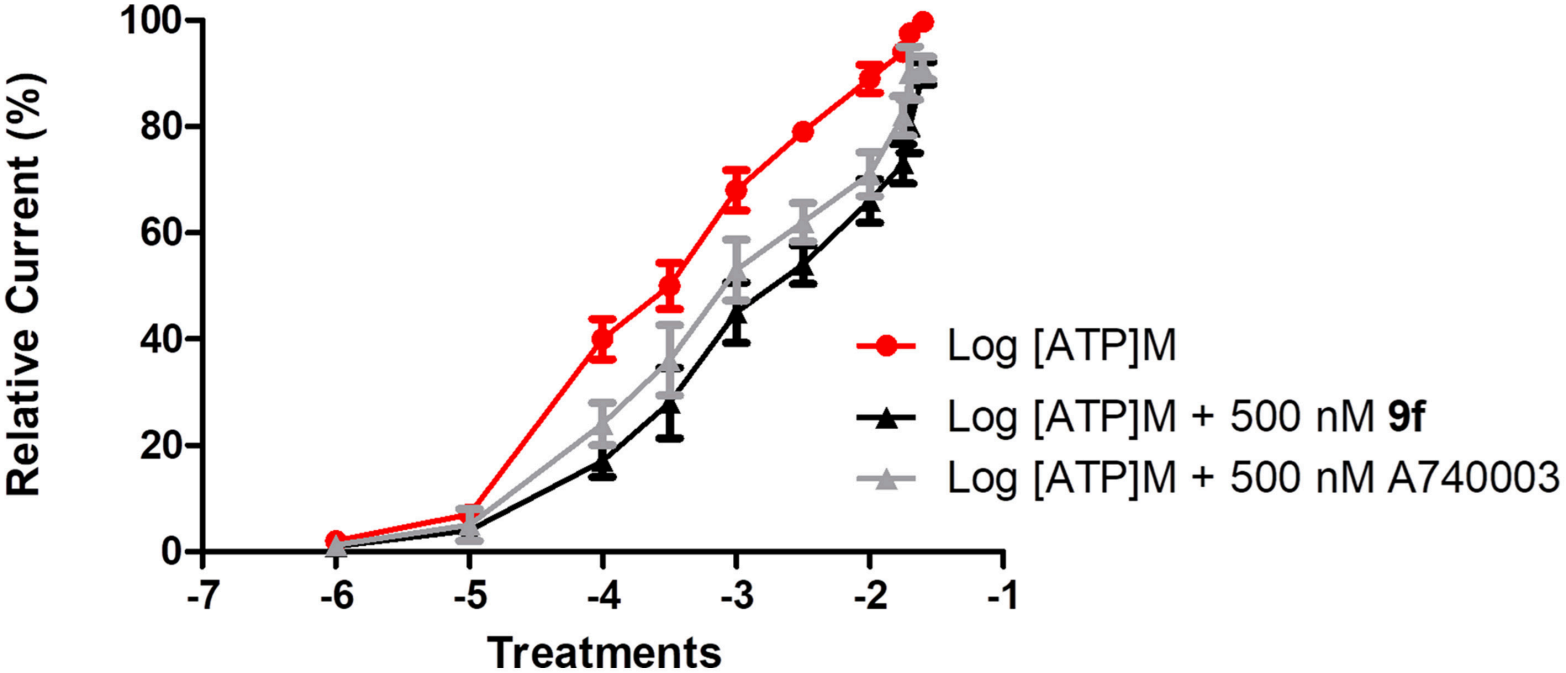
Inhibitory mechanism of compound **9f** on P2X7R. Inhibitory mechanism of action was evaluated using the whole-cell configuration. Ionic currents of mouse peritoneal macrophages stimulated with (•) adenosine triphosphate (ATP) concentrations alone, (■) ATP concentrations + a fixed concentration of 500 nM **9f** or ATP concentrations + a fixed concentration of 500 nM A750003. All experiments were performed at 30–37°C. Graphics are representative of three to four independent experiments.

To investigate the mechanism of action of the thiadiazole analogs, peritoneal macrophages treated with ATP concentrations (100 μM to 25 mM) alone or these same concentrations with a fixed concentration of **9f** (500 nM) were studied using the whole-cell configuration (Figure 1). The EC_50_ value for the ATP dose–response curve with a fixed **9f** concentration was higher than that for the ATP curve alone (Figure 1). The inhibition profile for **9f** was similar to that of A740003, which acts on the allosteric site of P2X7R (Honore et al., 2006; Lopez-Tapia et al., 2015; Karasawa and Kawate, 2016; Park and Kim, 2017).

The selectivity of **9f** for inhibiting P2X7R was confirmed through pharmacology. P2Y and P2X receptor antagonists were incubated for 10 min before P2X and P2Y agonist-induced [Ca^2+^]_i_ shift assays (Supplemental Table 2). The P2X1R agonist β,γ-meATP-induced intracellular Ca^2+^ mobilization was inhibited by 10 μM NF023; however, 100 μM **9f** did not reduce this effect in J774 cells. Undifferentiated PC12 pheochromocytoma cells preferentially express P2X2R (Sun et al., 2007). ATP-induced [Ca^2+^]_i_ shifts in PC12 cells were inhibited by 1 μM NF770; however, 10 μM A74003 and 100 μM **9f** did not inhibit these [Ca^2+^]_i_ shifts. The activity of the P2X3R agonist α,β-meATP was inhibited by 10 μM TNP-ATP; however, 100 μM **9f** did not reduce this effect. The NR8383 rat alveolar macrophage cell line expresses functional P2X4R, but functional P2X7R is not expressed (Bowler et al., 2003). ATP-induced [Ca^2+^]_i_ shifts potentiated by 1 μM ivermectin were inhibited by 1 μM 5BDBD; however, 10 μM A74003 and **9f** did not inhibit these shifts. ADP-induced [Ca^2+^]_i_ shifts mediated by P2Y1R activation were inhibited by 1 μM MRS 2179 in mouse peritoneal macrophages; however, A740003 and **9f** did not inhibit these effects. 2-S-UTP-activated [Ca^2+^]_i_ shifts mediated by P2Y2R were inhibited by 1 μM AR-C 118925XX in mouse peritoneal macrophages; however, A740003 and **9f** did not have inhibitory effects. MRS4062-induced [Ca^2+^]_i_ shifts mediated by P2Y4R were inhibited by 1 μM MRS2578 in mice peritoneal macrophages; however, A740003 and **9f** did not inhibit these shifts. UDPβS-activated [Ca^2+^]_i_ shifts mediated by P2Y6R were inhibited by 1 μM MRS 4162 in mouse peritoneal macrophages; however, A740003 and **9f** did not show inhibitory activity. NAADP-induced [Ca^2+^]_i_ shifts mediated by P2Y11R were inhibited by 1 μM NF340 in mouse peritoneal macrophages; however, A740003 and **9f** did not inhibit these shifts. 2MeSADP-activated [Ca^2+^]_i_ shifts mediated by P2Y12R were inhibited by 1 μM Ticagrelor in mouse peritoneal macrophages but were not inhibited by A740003 or **9f**. ADPβS-induced [Ca^2+^]_i_ shifts mediated by P2Y13R were inhibited by 1 μM MRS2211 in HEP2G cells, whereas A740003 and **9f** did not inhibit these shifts. UDP-glucose-activated [Ca^2+^]_i_ shifts mediated by P2Y14R were inhibited by 1 μM PPNT in mouse peritoneal macrophages but not by A740003 or **9f**.

#### Microsomal Stability, Solubility, and Permeability

Thiadiazoles (**9b**, **9c**, **9f**, and **11c**) exhibited solubility higher than 500 μM at pH values of 7.4 and 10. However, the solubility was reduced to 300 μM at acidic pH values.

All selected thiadiazoles exhibit stability in mouse and human microsomes. Among these compounds, analog **11c** was the most stable in mouse and human microsomes (Table 5). Compared to the controls and **9f** and **11c** analogs, analogs **9b** and **9c** showed moderate permeability in Caco-2 cells (Table 5).

**Table 5:** Liver microsomal stability and Caco-2 cell permeability of thiadiazole derivatives.

**Compounds**	**LM stability (a) mouse**	**LM stability (a) human**	**Caco-2 (b)**
9b	14.3	26.4	50.1 ± 1.1
9c	11.6	18.6	56.1 ± 2.1
9f	26.7	39.5	73.4 ± 3.3
11c	38.51	46.7	77.9 ± 1

*(a) Mouse and human liver microsome (LM) stability. Data reported as CLint (μl/minutes/mg protein)*.

*(b) Apparent permeability values (Papp) measured using low-permeability and high-permeability absorption compounds, vinblastine, and propranolol, respectively, as a reference. Data are reported in 10^6^ cm/s. These values indicate an apical to basolateral (A - B) direction. Values are mean ± standard error of three experiments*.

Thiadiazoles **9b** and **9c** showed higher log D_7.4_ values than analogs **9f** and **11c** (Table 6). The increased solubility of **9f** and **11c** in aqueous solution may be related to their microsomal stability, which is higher than that of **9b** and **9c**. Additionally, the higher aqueous solubility and microsomal stability possibly increase the Caco-2 permeability of these thiadiazoles (Table 6).

**Table 6:** Log D_7.4_ of thiadiazole derivatives.

**Compounds**	**Log D_**7.4**_ (a)**
9b	−1.2 ± 0.1
9c	−0.9 ± 0.1
9f	−3.7 ± 0.2
11c	−4.2 ± 0.4

*(a) Results are the average of three experiments, and in all cases, individual log D values were within ± 0.3 log units of the average log D propranolol HC*.

#### *In vivo* Anti-inflammatory Activity

**Figure 2:**
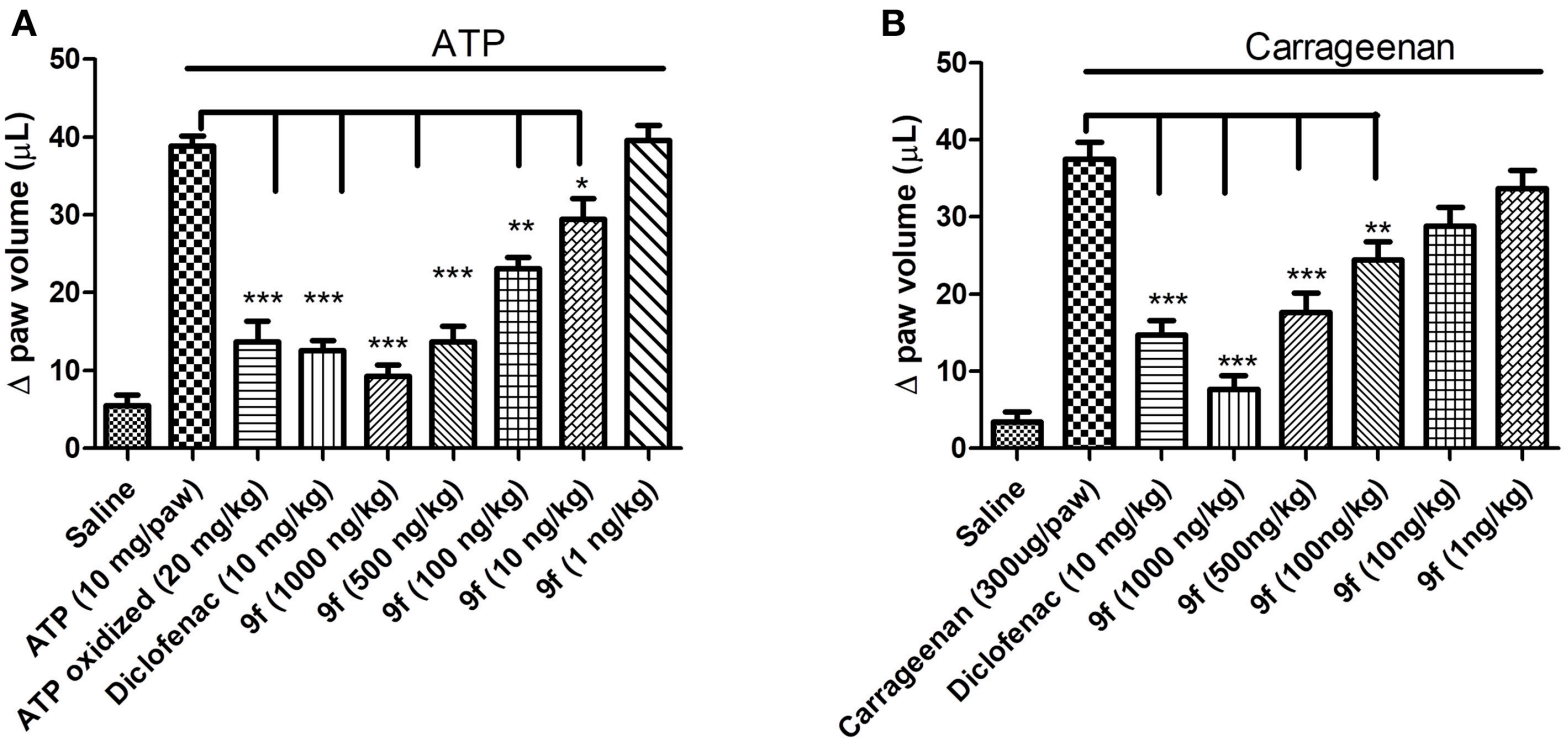
*In vivo* inhibition of paw edema formation by derivative **9f** in mice. The mice were pretreated for 1 h with oxidized ATP **(A)**, diclofenac **(A,B)**, or derivative **9f (A,B)** in crescent concentrations using the intraperitoneal pathway. ATP **(A)** or carrageenan **(B)** was applied (intraplantar), and the paw edema was measured after 30 or 60 min, respectively. The black line above the bars represents the stimulation with ATP or carrageenan. These results are representative of three to five experiments that were performed on distinct days. ^*^p < 0.05, ^**^p < 0.01, ^***^p < 0.001.

Based on the P2X7R-mediated IL-1β release inhibition results *in vitro*, the activity of analog **9f** against the inflammatory response was evaluated using ATP- and carrageenan-induced paw edema. Oxidized ATP, sodium diclofenac, and analog **9f** were intraperitoneally administered 1 h before ATP treatment in the paw. For carrageenan, only oxidized ATP was not used. Paw edema formation was measured 30 min after ATP application or 60 min after carrageenan application. Analog **9f** dose-dependently decreased ATP- and carrageenan-induced edema (Figures 2A,B). When the higher dose was used, the derivative **9f** caused greater inhibition than oxidized ATP (Figure 2A) and diclofenac (Figures 2A,B). These results are similar to those from a previous publication testing the P2X7R activity of triazoles and naphthoquinone molecules (Gonzaga et al., 2017; Faria et al., 2018).

### In Silico

#### ADMET Analysis

The toxicological parameters predicted by the Osiris® program indicated a low probability of mutagenic and tumorigenic effects of the four most potent thiadiazole derivatives: **9b**, **9c**, **9f**, and **11c**. Moreover, irritable effects and interference with the biological reproduction process were not identified. The toxicological *in silico* results are promising; however, *in vitro* and *in vivo* analyses in future studies will be necessary to confirm these results.

The pharmacokinetic parameters of compounds **9b**, **9c**, **9f**, and **11c** predicted using the program ADMET Predictor® indicate that compared to known anti-inflammatory drugs, such as diclofenac, ibuprofen, and naproxen, they present similar lipophilicity (LogP) and blood–brain barrier (BBB) permeability (Table 7) (Gonzaga et al., 2017).

**Table 7:** *In silico* analysis of ADMET parameters of thiadiazole derivatives compared to drugs in therapeutic use.

**Compound**	**LogP**	**Peff**	**BBB**	**FaSSGF**	**FaSSIF**	**PrUnbnd**
9b	2.44	3.64	High	0.196	0.00986	8.33
9c	2.31	3.75	High	0.507	0.0182	12.54
9f	2.61	4.55	High	0.0692	0.0168	3.85
11c	3.29	7.71	High	0.33	0.0192	6.42
Diclofenac	3.58	6.4	High	0.0127	0.144	0.38
Ibuprofen	2.82	6.52	High	0.0199	0.52	2.1
Naproxen	2.35	6.44	High	0.0607	0.61	1.33

Although commercial anti-inflammatory drugs are predicted to be more preferentially solubilized in the intestinal fluid (FaSSIF) than compounds **9b**, **9c**, **9f**, and **11c**, thiadiazole derivative **11c** is predicted to have the highest human jejunal permeability (Peff). Regarding the binding degree to blood plasma proteins (PrUnbnd), compounds **9b**, **9c**, **9f**, and **11c** are predicted to present a more considerable number of unbound molecules than commercial anti-inflammatory drugs. This free portion in the blood may indicate a higher diffusion to tissues, resulting in a higher potency (Table 7).

#### Structure and Dynamics of the Human and the Mouse P2X7R in the Free and Bound Systems

Molecular docking indicated that compound **9f** interacts with the same molecular area in both human and murine P2X7R. The dynamic behavior of human (hP2X7R) and mouse (mP2X7R) P2X7R before and after binding to **9f** (the most active compound evaluated in our work) was evaluated during 200 ns of MD simulations of the following P2X7R systems in membrane-aqueous medium: hP2X7R_APO_, mP2X7R_APO_ (free P2X7R), hP2X7R_9f_, and mP2X7R_9f_ (**9f**–P2X7R complex).

The analysis of the P2X7R SS in the molecular systems hP2X7R_APO_, mP2X7R_APO_, hP2X7R9f, and mP2X7R_9f_ showed that the structural variation was acceptable, maintaining molecular stability during the entire 200 ns of MD simulation time. The inhibitor binding does not change the receptor fold (Supplemental Figures 1A–D).

**Figure 3:**
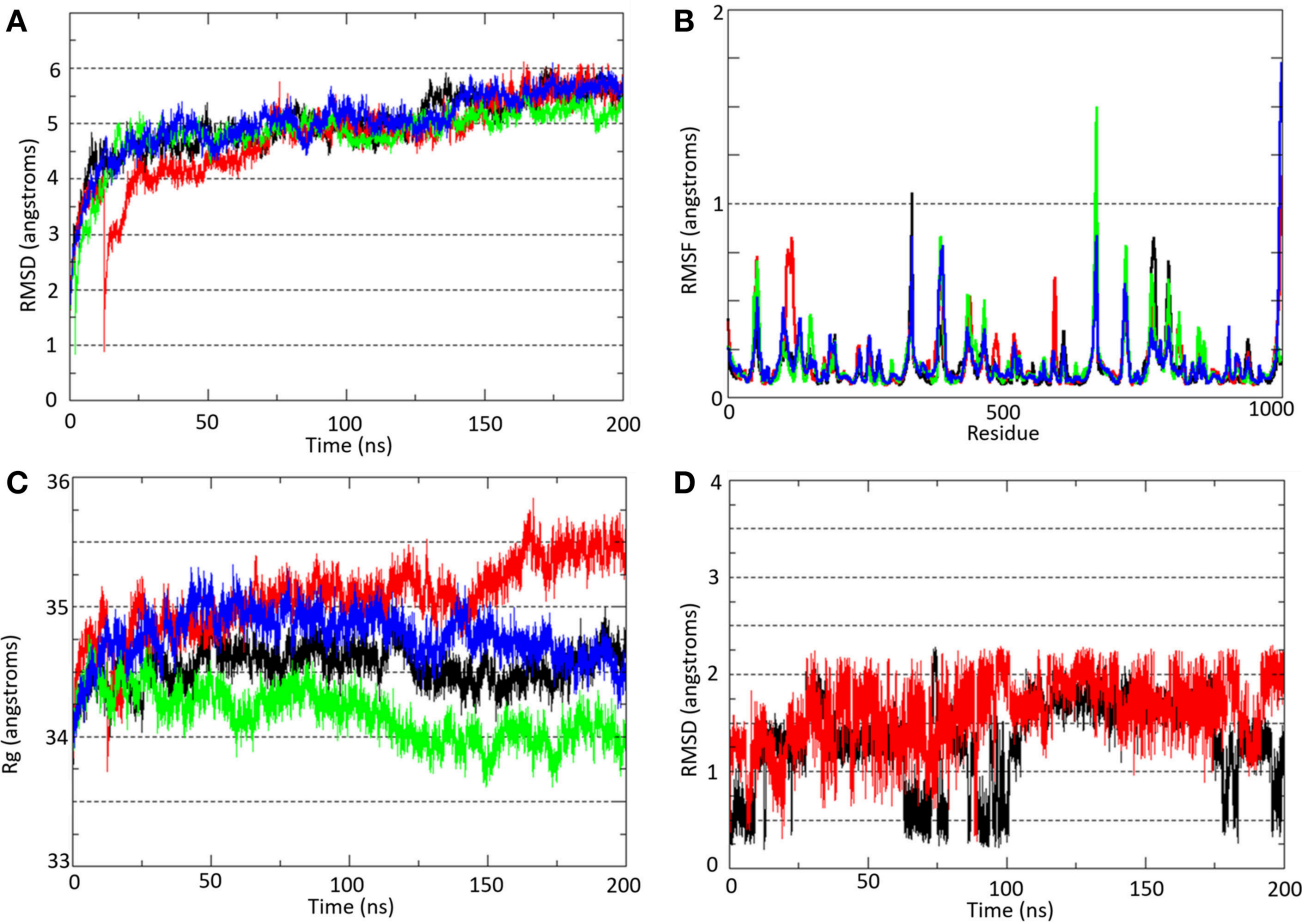
**(A)** Root mean square deviation (Cα-RMSD) and **(B)** root mean square fluctuation (Cα-RMSF) analyses of all Cα-atoms of P2X7R in the hP2X7R_APO_ (black line), hP2X7R_9f_ (red line), mP2X7R_APO_ (green line), and mP2X7R_9f_ (blue line) systems. **(C)** Radius of gyration (Rg) analysis of the hP2X7R_APO_ (black line), hP2X7R_9f_ (red line), mP2X7R_APO_ (green line), and mP2X7R_9f_ (blue line) systems. **(D)** Root mean square deviation (Cα-RMSD) analysis of inhibitor **9f** in the hP2X7R_9f_ (red line) and mP2X7R_9f_ (blue line) systems. The Cα-RMSD and Rg analyses were carried out throughout the entire simulation, while the Cα-RMSF analysis showed the residue fluctuation during the last 150 ns of simulation.

At ~50 ns, the molecular systems hP2X7R_APO_ (black line), hP2X7R_9f_ (red line), mP2X7R_APO_ (green line), and mP2X7R_9f_ (blue line) achieve stability, presenting average RMSD values between 4.5 and 6.0 during the MD simulation (Figure 3A). These results were obtained by comparing the root mean square deviation values of all P2X7R Cα-atoms (Cα-RMSD) relative to the initial structures.

Over the last 150 ns of MD simulation (Figure 3B), the root mean square fluctuations of all P2X7R Cα-atoms (Cα-RMSF) for the molecular systems hP2X7R_APO_ (black line), hP2X7R_9f_ (red line), mP2X7R_APO_ (green line), and mP2X7R_9f_ (blue line) were calculated to analyze the structure stability in each segment of the receptor. The plot with the Cα-RMSF results shows that there is no significant variation in the receptor structure fluctuation, with differences among their fluctuations below 1.0 Å. The only exception is the transmembrane helix of the C chain in the hP2X7R_9f_ and mP2X7R9f systems (the region between Gln306 and Cys337 in both receptors), which presents higher RMSF values than those of the apo systems. Thus, the binding of inhibitor **9f** to the human and mouse receptors seems to increase the fluctuation of this transmembrane helix by approximately 1.7 Å.

The radius of gyration (Rg) results (Figure 3C) indicate only slight increases of 1.0 and 0.5 Å for the hP2X7R_9f_ and mP2X7R_9f_ systems (red and blue lines) in comparison with the hP2X7R_APO_ and mP2X7R_APO_ systems (black line), respectively, during the last 50 ns of simulation. Thus, these results suggest that the presence of **9f** decreases the compactness of both receptors and increases their Rg values. Additionally, the RMSD analysis of the **9f** structure shows that this blocker moved away from its initial position by approximately 2.0 Å in both the hP2X7_9f_ and mP2X7_9f_ systems (Figure 3D) during the last 150 ns of simulation.

#### Analysis of Hydrogen Bonding Interactions Between the P2X7R and the 9f Inhibitor

**Figure 4:**
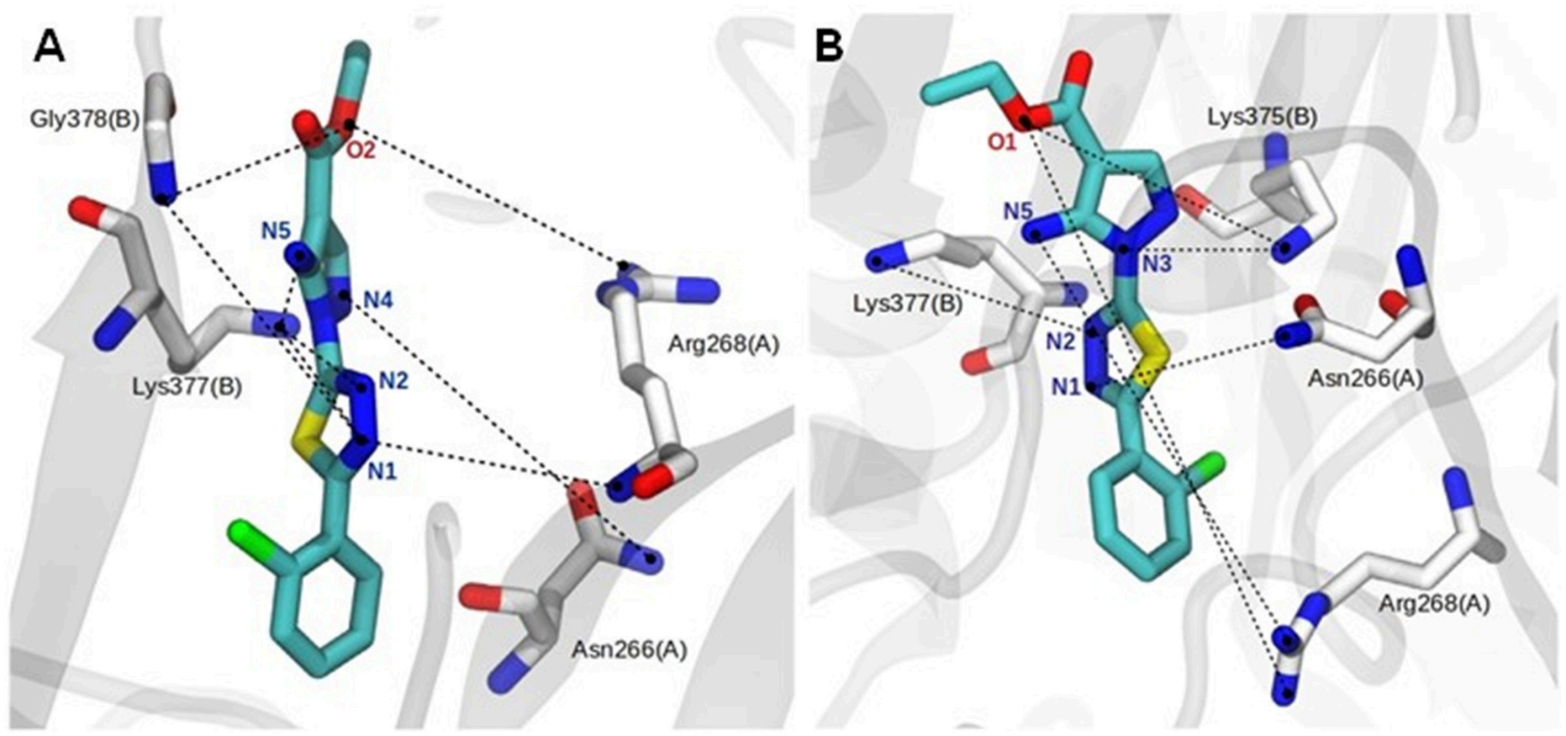
Close view of the main hydrogen bonds (H-bonds, black dotted lines) between the agonist and human **(A)** and mouse **(B)** P2X7R residues during the last 150 ns of molecular dynamics simulation in aqueous solution. Analog **9f** and P2X7R residues are represented as stick models and colored by atom (carbon, gray, or white; nitrogen, blue; oxygen, red; fluorine, pink), and all hydrogen atoms are omitted for clarity.

The binding mode of ligand **9f** showed intermolecular interactions by hydrogen bonds (H-bonds), mainly with the Asn266 and Arg268 residues of the A chain and the Lys375, Lys377, and Gly378 residues of the B chain (Figures 4A,B). In the hP2X7R_9f_ system (Figure 4A), the N1 atom of compound **9f** forms H-bonds with the backbone NH_2_ group of the Arg268 (chain A; lifetime = 14.71 ns) and Gly378 residues (chain B; lifetime = 17.03 ns) and with the side chain of Lys377 (chain B; lifetime = 19.48 ns), which is the most persistent H-bond in this molecular system. The N2 atom of this compound interacts with the side-chain NH_2_ group of Lys377 (chain B; lifetime = 12.09 ns), while the N4 and N5 atoms generate H-bonds with the side-chain NH_2_ group of Asn266 (chain A; lifetimes = 4.39 ns) and Lys377 (chain B; lifetimes = 5.17 ns), respectively. The ester O2 atom of compound **9f** also interacts with the backbone NH_2_ group of Gly378 (chain B; lifetime = 6.86 ns) and with the guanidinium group of Arg268 (chain A; lifetime = 5.45 ns; Figure 4A).

The complex formed between the mouse receptor (mP2X7R) and compound **9f** presents similar interactions as those described for the hP2X7R_9f_ system (Figure 4A). Thus, the N1 atom of compound **9f** makes an H-bond with the side-chain NH_2_ group of Asn266 (chain A; lifetime = 12.08 ns), while the N2 atom of this ligand interacts with the side-chain NH_2_ group of Lys375 (chain B; lifetime = 1.16 ns). The N3 and N5 atoms interact with the side chain of Lys377 (chain B; lifetime = 3.99 ns) and with the guanidinium group of Asn268 (chain A; lifetime = 8.95 ns), respectively. In addition, the ester O1 atom of compound **9f** interacts with the side-chain NH_2_ group of Lys377 (chain B; lifetime = 41.86 ns), which is the most persistent interaction in this system, and with the guanidinium group of Arg268 (chain A; lifetime = 2.09 ns; Figure 4B). Other intermolecular H-bonds are also observed in the **9f**–hP2X7R and **9f**–mP2X7R complexes but in the range of only a few picoseconds of MD simulation time.

#### Cross-Correlation Map Analysis in the Free and Bound Protein Systems

**Figure 5:**
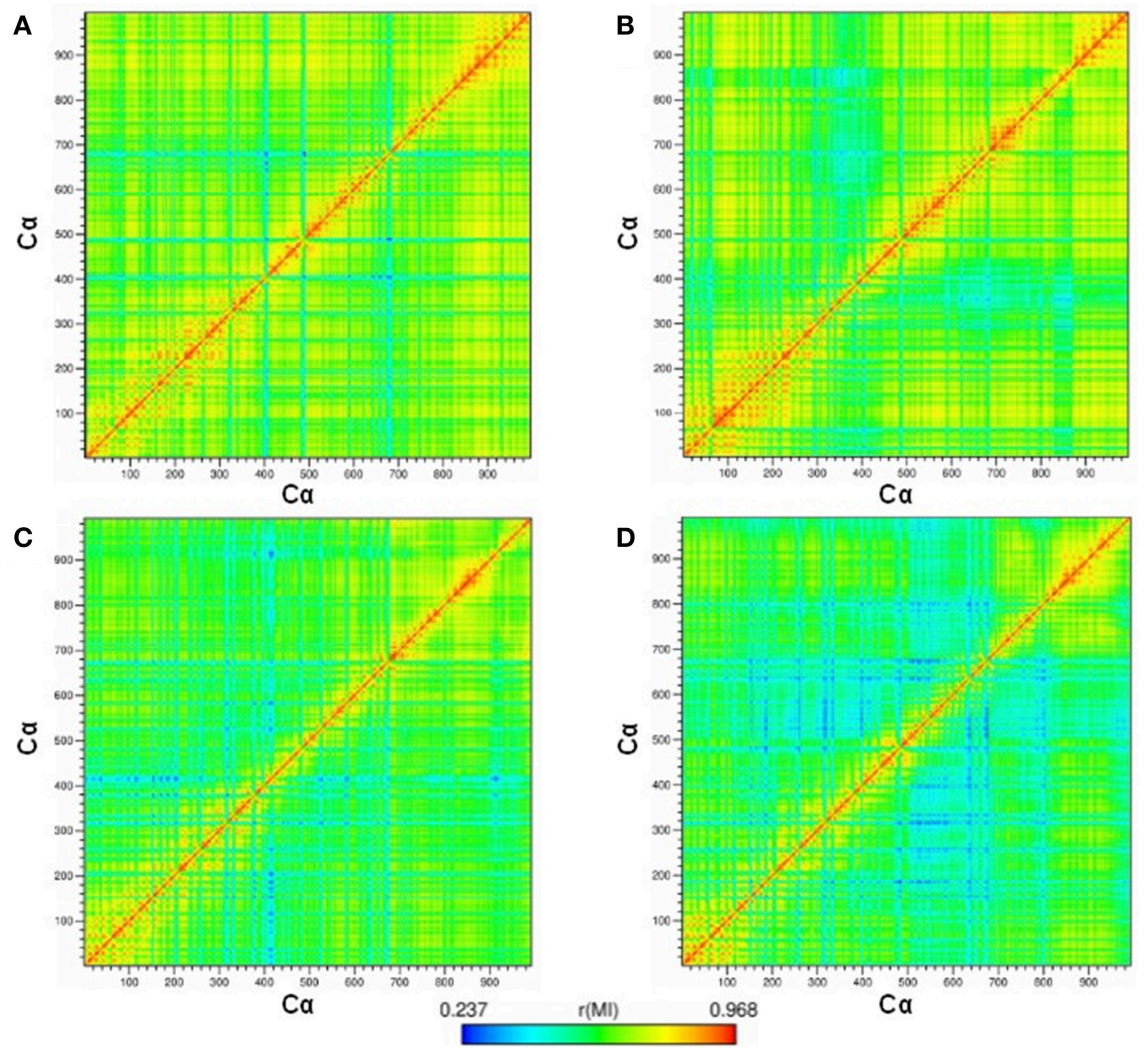
Maps of the correlated motions among the P2X7R Cα-atoms in the **(A)** hP2X7R_APO_ (free receptor), **(B)** hP2X7R_9f_ (antagonist-bound receptor), **(C)** mP2X7R_APO_, and **(D)** mP2X7R_9f_ systems during the last 150 ns of simulation time. Note: The strength of the computed correlation between two respective CRZ Cα-atoms is color coded (see the color bar on the bottom), where highly correlated motions are in red and poorly correlated motions are in blue.

**Figure 6:**
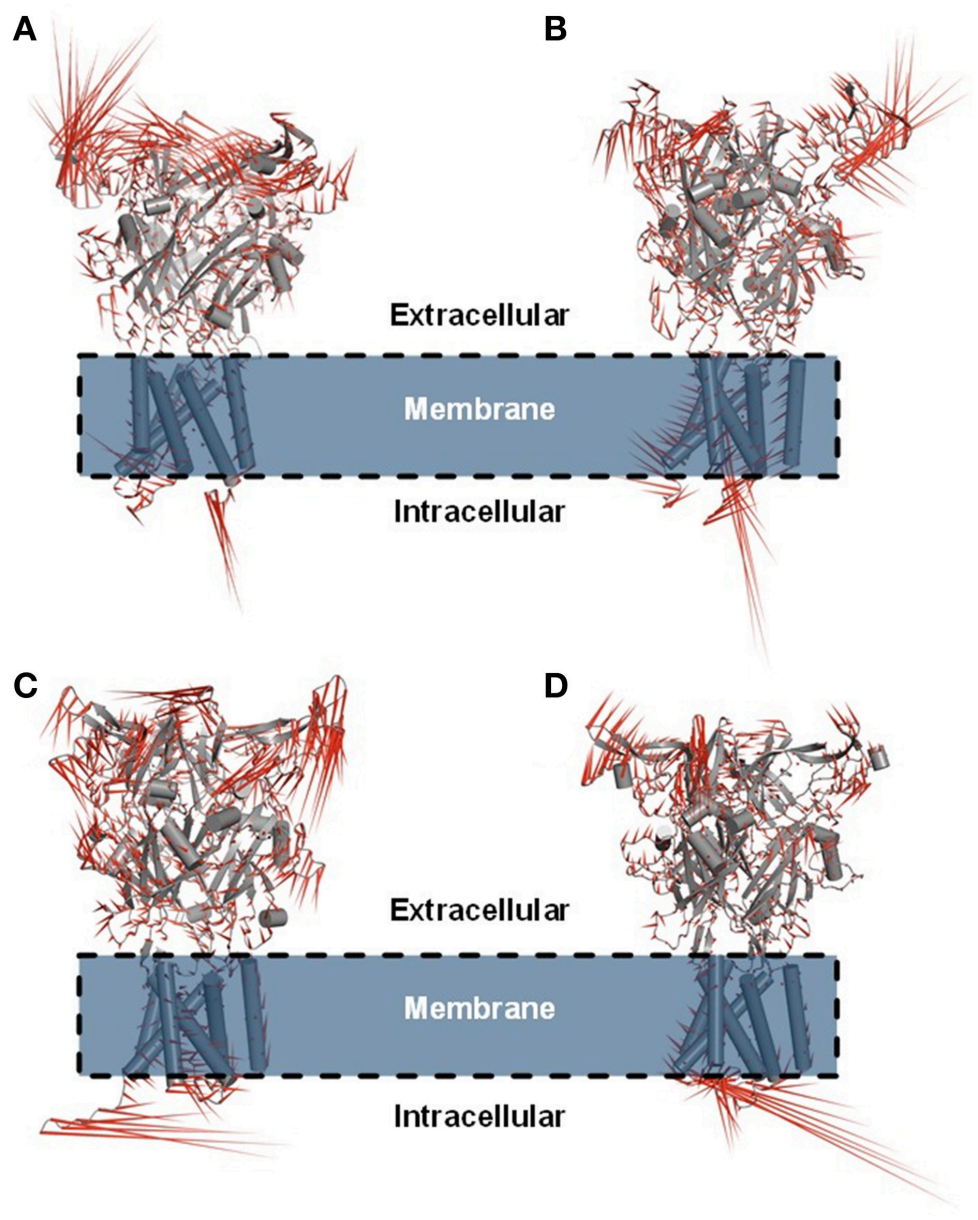
Porcupine plot showing the main concerted motions of the hP2X7R and mP2X7R structures in the hP2X7R_APO_ **(A)**, hP2X7R_9f_ **(B)**, mP2X7R_APO_ **(C)**, and mP2X7R_9f_ **(D)** systems during the last 150 ns of molecular dynamics simulation.

The correlation between the motions of the Cα-atom residues was evaluated to observe the conformational modifications of the receptor before and after binding to compound **9f**. Thus, cross-correlation map analysis was performed for all systems (Figures 5A–D). This approach aids in obtaining information concerning the correlation between the fluctuations of the positions of the Cα-atom residues. The comparison between the hP2X7R_APO_ and hP2X7R_9f_ systems (Figures 5A,B) shows that the interaction of **9f** with hP2X7R decreases the correlation, mainly between the Gly1(chain A)–Cys162(chain C) domain [1–800 Cα] and the Ala146(chain C)–Gly260(chain C) regions [820–880 Cα], as well as between the Ser260(chain A)–Cys103(chain B) [260–440 Cα] and Thr163(chain B)–Cys126(chain C) regions [500–800 Cα] (Figure 5B). Similarly, when we compare the mP2X7R_APO_ and mP2X7R_9f_ systems (Figures 6C,D), the binding of **9f** to mP2X7R reveals an expressive reduction in correlations between the Ala140(chain A)–Thr123(chain B) [140–460 Cα] and Pro143(chain B)–Phe6(chain C) [480–680 Cα] regions and between the Arg180(chain A)–Phe6(chain C) (180–680 Cα) and Asp106(chain C)–Ala146(chain C) regions (780–820 Cα) of mP2X7R (Figure 5D). This finding suggests that the binding of the inhibitor **9f** to both receptors (hP2X7R and mP2X7R) interferes with the correlated motions of the P2X7R structure. This effect is probably associated with the initial steps of the mechanism of action of inhibitor **9f**.

#### Principal Component Analysis in the Free and Bound P2X7R Systems

Principal component analysis (PCA) was also carried out on the last 150 ns of simulation to better characterize the nature of the collective motions of hP2X7R and mP2X7R in the simulated systems. PCA focuses on these dominant motions, represented as porcupine projections (Figures 6A–D). The P2X7R backbone movement projections show vectors, which indicate the direction and extent of the motion. The porcupine projection analysis indicates that the binding of compound **9f** reduces the movements of the extracellular motif (head, upper body, right flipper, left flipper, dorsal fin, and lower body regions) of human and mouse P2X7R (Figures 6A,B). In addition, hP2X7R, when complexed to **9f** (Figure 6B), seems to lose part of its SS of the head region, which is not evident in mP2X7R (Figure 6D). The movements found in apo molecular systems may be necessary for the biological function of P2X7R. Thus, the inhibitor **9f** binding mode with intermolecular H-bond interactions seems to reduce the extracellular motions, which are typically sampled in the apo form, thereby inactivating this receptor (Figures 6A–D).

## Discussion

The hypothetical relevance of P2X7R in health and disease has been promoted in diverse studies on the generation of selective P2X7 antagonists with therapeutic efficacy in animal models of disease and clinical assays.

A large variety of P2X7 ligands effectively inhibit human, rat, and mouse P2X7R in a potent manner, such as compounds A-740003 and JNJ47965567 (Donnelly-Roberts et al., 2009; Bhattacharya et al., 2013; Lord et al., 2014; Jacobson and Muller, 2016; Swanson et al., 2016). Despite the restricted availability of these newer inhibitors, a large number of scientific groups are still using traditional inhibitors (oxidized ATP and BBG).

Additionally, the inhibitors AZD9056, CE-224535, and GSK1482169 have proven to be safe and well-tolerated in humans; however, their clinical development has been discontinued because of insufficient clinical efficacy in Phase II studies for rheumatoid arthritis (Pevarello et al., 2017).

A possible explanation for this limited efficacy in clinical rheumatoid arthritis assays is deficient drug pharmacokinetic and pharmacodynamic properties (Arulkumaran et al., 2011; Pevarello et al., 2017). Based on this explanation, some companies are testing anti-P2X7R anti-bodies in preclinical tests (Danquah et al., 2016) or clinical assays (Gilbert et al., 2017).

Therefore, the lack of availability or high cost of diverse inhibitors to many laboratories (Pevarello et al., 2017) and the lack of description about the effects of many of these compounds on recombinant murine variants limit the study of P2X7 in pathological conditions. Thus, the study of new substances of chemical classes poorly explored as P2X7R antagonists is still necessary. Chemical classes such as pyrazoles and thiadiazole have been rarely or not at all studied as P2X7R inhibitors, respectively.

Basically, only in 2008 did the patent WO2008125600A2 (Beswick et al., 2008) evaluate pyrazoles as P2X7R inhibitors. Pyrazole analogs were synthesized by replacing a heterocyclic core with an imidazole. Compound **10** exhibited potent inhibitory effects (pIC_50_ 7.4), low *in vitro* metabolism, and high solubility in aqueous medium (Gleave et al., 2010).

Compounds derived from 2-Cl-5-heteroaryl-benzamide exhibited antagonistic action against the P2X7R. However, these analogs showed poor metabolic stability and low volume of distribution in tissues Vd (ss). *In silico* optimization led to the identification of pyrazole **39**, which exhibited excellent potency and oral bioavailability but low Vd (ss). Therefore, analogs **40** and **41** incorporated amine groups and maintained the elevated potency and improved Vd (ss) only for i.v. administration (Subramanyam et al., 2011). However, these compounds exhibited poor oral absorption.

Thiadiazole **9f** showed elevated potency and selectivity for inhibiting P2X7R function *in vitro*, low toxicity to mammalian cells, good solubility in aqueous solutions, low metabolic stability, and potent inhibition of acute inflammation. In comparison to other molecules described by our group (Gonzaga et al., 2017; Faria et al., 2018; Pacheco et al., 2018) or various other groups and companies (Pevarello et al., 2017), **9f** showed compatible pharmacological properties. Therefore, this thiadiazole analog may be a good candidate to inhibit P2X7R with therapeutic activity in humans.

In the present work, MD simulation was used to increase the details and precision of the analysis of the intermolecular interactions between thiadiazole **9f** and the P2X7 protein. It is possible to observe that compound **9f** is stabilized by hydrogen bonding interactions, mainly with Arg, Asn, and Lys residues. Previous studies involving human P2X7 models have studied inhibitors of the naphthoquinone (Faria et al., 2018) and triazole (Gonzaga et al., 2017) classes without the aid of MD simulations, and therefore, a more detailed comparison between these classes of inhibitors employing MD simulation should also be performed. However, it is possible that these different classes of inhibitor compounds have affinity for different residues. While thiadiazole derivative **9f** interacts mainly by hydrogen bonds, the naphthoquinone-derived P2X7 inhibitors may act by π-stacking and hydrophobic interactions with residues such as Tyr, Phe, and Val.

## Conclusion

In summary, the synthesis of a series of novel thiadiazole derivatives targeting P2X7R led to the discovery of potent inhibitors, such as **9f** (IC_50_ = 16 nM). The inhibitory effects of these compounds were higher than those of the reference compounds BBG (IC_50_ = 1.28 μM) and A730003 (IC_50_ = 0.108 μM) using the dye uptake assay. Moreover, derivative **9f** showed acceptable *in vitro* inhibitory action against P2X7R; pharmacokinetic, pharmacodynamic, and toxicological properties *in silico*; and anti-inflammatory activity *in vivo*. The potent P2X7R inhibitor discovered in this study might provide an opportunity for anti-inflammatory drug discovery targeting the receptor.

Additionally, 200 ns of MD simulation was carried out to study the structure and dynamic behavior of the human and mouse P2X7Rs in a complex with compound **9f**. First, by comparing the conformational changes between the hP2X7R_APO_, mP2X7R_APO_ (free receptors), hP2X7R_9f_, and mP2X7R_9f_ (bound receptors) molecular systems in aqueous medium during the MD simulation, it was found that the binding of **9f** reduces the movements of the extracellular motif (head, upper body, right flipper, left flipper, dorsal fin, and lower body regions) of the human and mouse P2X7R.

Additionally, the binding mode of blocker **9f** with P2X7R through intermolecular H-bond interactions with key residues (mainly with Asn266 and Arg268 residues of the A chain and Lys375, Lys377, and Gly378 residues of the B chain) can reduce the extracellular movements of the receptor. Then, the cross-correlation analysis shows evidence that the binding of inhibitor **9f** to P2X7R decreases the correlated motions found in apo systems. Therefore, these findings will provide understanding of the conformational dynamics in the P2X7R inactivation process, where the compounds that reduce the extracellular movements of the receptor represent putative P2X7R blockers and, consequently, an alternative treatment for purinergic disorders.

## Ethics Statement

Our protocols adhered to the Ethical Principles in Animal Experimentation adopted by the Brazilian College of Animal Experimentation and approved by the FIOCRUZ Research Ethics Committee (number L039- 2016). Male mice (Swiss Webster) peritoneal macrophages were collected from peritoneal cavity lavage, platted for 24 h before dye uptake assay, whole cell patch clamp experiments, and IL-1β release assay.

## Author Contributions

DG, LP, HS, CR, and JM performed the experiments (chemical synthesis). JS, RF, FO, and GP performed the experiments (biological assays). RF, DG, LP, LH, MB, and NB wrote the paper. NvR, MB, HC, CR, RL, and LH performed the *in silico* studies.

### Conflict of Interest Statement

The authors declare that the research was conducted in the absence of any commercial or financial relationships that could be construed as a potential conflict of interest.
