# Supplementary material for: Synthesis, Biological Evaluation, and Molecular Modeling Studies of New Thiadiazole Derivatives as Potent P2X7 Receptor Inhibitors
Source: Front Chem. 2019 Apr 30;7:261. doi: 10.3389/fchem.2019.00261 (PMC6511888; doi:10.3389/fchem.2019.00261)
Supplement: Supplementary file 1 [file Table_1.pdf]

Supplemental Table S1. Composition of the lipid used to construct the membrane bilayer

Lipid name	Type	Value for each leaflet
cholesterol	-	19
ERG	-	0
PC (phosphatidylcholine)	POPC	69
PE (phosphatidylethanolamine)	POPE	38
PS (phosphatidylserine)	POPS	3
PI (phosphatidylinositol)	SAPI	15
SM (sphingo)	PSM	3
