# Supplementary material for: Synthesis, Biological Evaluation, and Molecular Modeling Studies of New Thiadiazole Derivatives as Potent P2X7 Receptor Inhibitors
Source: Front Chem. 2019 Apr 30;7:261. doi: 10.3389/fchem.2019.00261 (PMC6511888; doi:10.3389/fchem.2019.00261)
Supplement: Supplementary file 2 [file Table_2.pdf]

Supplemental Table S2. The 9f selectivity to inhibit P2X7R.

P2R subtype	P2R Agonist (%)*	P2R Agonist + Selective P2R subtype antagonist	P2R Agonist + A740003	P2R Agonist + 9f	Cell type
P2X1R	10 µM β,γ-me ATP (18 ± 2)	10 µM β,γ-me ATP + 10 µM NF023 (1.2 ± 0.31)	10 µM β,γ-me ATP (17 ± 3)	10 µM β,γ-me ATP (19 ± 2)	J774 cells
P2X2R	10 µM ATP (31 ± 3)	10 µM ATP + 1 µM NF770 (4.1 ± 1)	10 µM ATP (29.6 ± 5)	10 µM ATP (30.1 ± 4)	PC12 cells
P2X3R	10 µM α,β-me ATP (14 ± 1)	10 µM α,β-me ATP + 10 µM TNP-ATP (1 ± 0.3)	10 µM α,β-me ATP (11 ± 5)	10 µM α,β-me ATP (12 ± 3)	Mice peritoneal macrophages
P2X4R	10 µM ATP (36 ± 5) 10 µM ATP + 1 µM ivermectin (40 ± 5)	10 µM ATP + 1 µM ivermectin + 10 µM 5BDBD (7 ± 2)	10 µM ATP + 1 µM ivermectin (40 ±7)	10 µM ATP + 1 µM ivermectin (42 ± 4)	NR8383 cells
P2Y1R	10 µM ADP (12 ± 1)	10 µM ADP + 1 µM MRS 2179 (0.7 ± 0.2)	10 µM ADP (11 ± 3)	10 µM ADP (11 ± 2)	Mice peritoneal macrophages
P2Y2R	10 µM 2-S-UTP (52 ± 1)	10 µM 2-S-UTP + 1 µM AR-C 118925XX (2.4 ± 1)	10 µM 2-S-UTP (51 ± 3)	10 µM 2-S-UTP (51 ± 2)	Mice peritoneal macrophages
P2Y4R	10 µM MRS4062 (19 ± 5)	10 µM MRS4062 + MRS2578 (1.1 ± 0.5)	10 µM MRS4062 (21 ± 4)	10 µM MRS4062 (20 ± 3)	Mice peritoneal macrophages
P2Y6R	10 µM UDPβS (14 ± 2)	10 µM UDPβS + 10 µM MRS4162 (11 ± 4)	10 µM UDPβS (15 ± 3)	10 µM UDPβS (14 ± 3)	Mice peritoneal macrophages
P2Y11R	10 µM NAADP (23 ± 5)	10 µM NAADP + 10 µM NF340 (3.6 ± 1)	10 µM NAADP (26 ± 4)	10 µM NAADP (26 ± 4)	Mice peritoneal macrophages
P2Y12R	10 µM 2MeSATP (37 ± 3)	10 µM 2MeSATP + 1 µM Ticagrelor (2.1 ± 0.7)	10 µM 2MeSATP (41 ± 7)	10 µM 2MeSATP (38 ± 5)	Mice peritoneal macrophages
P2Y13R	10 µM ADPβS (30 ± 4)	10 µM ADPβS + 1 µM MRS2211 (4 ± 1)	10 µM ADPβS (31 ± 3)	10 µM ADPβS (31 ± 5)	HEP2G cells
P2Y14R	10 µM UDP-glucose (22 ± 2)	10 µM UDP-glucose + 1 µM PPNT (1.5 ± 0.8)	10 µM UDP-glucose (24 ± 5)	10 µM UDP-glucose (21 ± 4)	Mice peritoneal macrophages
