# Supplementary material for: Synthesis, Biological Evaluation, and Molecular Modeling Studies of New Thiadiazole Derivatives as Potent P2X7 Receptor Inhibitors
Source: Front Chem. 2019 Apr 30;7:261. doi: 10.3389/fchem.2019.00261 (PMC6511888; doi:10.3389/fchem.2019.00261)
Supplement: Supplementary file 3 [file Data_Sheet_1.docx]

Supplemental Materials and Methods

**1. Chemistry**

All reagents and solvents used were analytical grades. Fourier transform infrared (FT-IR) absorption spectra were recorded on a Shimadzu mode IR Prestige-21 spectrophotometer. The melting points (m.p.) were measured employing the Büchi model B-545. The BRUKER Avance instrument was used to obtain the spectras of ^1^H, ^13^C and ^19^F nuclear magnetic resonance (NMR) in frequences of 400.00,100.00 and 376.00 MHz MHz respectively. The chemical shifts (δ) are presented in ppm, and the coupling constants (*J*) are stated in Hertz. High- Resolution Mass Spectrometry (HRMS) spectras were made by an LC-MS Bruker Daltonics MicroTOF. The reactions were accompanied by TLC (thin layer chromatography) silica gel F-254 glass plate (20 x 20 cm).

**1.1 General procedure for preparation of ethyl 5-amino-1-(1,3,4-thiadiazol-2-yl)-1*H*-pyrazole-4-carboxylate (9a-h)**

The respective 2-hydrazinyl-1,3,4-thiadiazole (**7**) (2 mmol) and (ethoxymethylene)malononitrile (**8a**) or ethyl (ethoxymethylene)cyanoacetate (**8b**) were dissolved in 40 mL of ethanol and the mixture was stirred and refluxed for 2-4 h. The reactions were accompanied by TLC (CHCl_3_/MeOH 9:1). After detecting the end of the reaction, the reaction medium was poured onto ice-cold water (50 mL). The product was precipitated, filtered, washed with cold water and recrystallized using the solvent mixture ethanol/water (3:1) to produce 9a-h at 55-91% yield.

5-amino-1-(5-phenyl-1,3,4-thiadiazol-2-yl)-1*H*-pyrazole-4-carbonitrile **(9a)**. Yield: 85%. brown solid. MP: 234-235 ºC. IR (cm^-1^): 3398; 3312; 2364; 2342; 2219; 1708; 1628; 1579; 1560; 1508; 1459; 1425; 1360; 1221; 1092; 982; 932; 763; 688; 669; 616; 589; 578; 559; 542; 533. ^1^H NMR (400 MHz, DMSO-d_6_, TMS, *δ* in ppm): 7.55-7.60 (m, 3H, C-3´´, C-4´´ and C-5´´); 7.98-8.00 (m, 2H, C-2´´ and C-6´´); 8.07 (s, 1H, H-3´); 8.11 (s, 2H, NH_2_). ^13^C NMR (100 MHz, DMSO-d_6_, TMS, *δ* in ppm): 73.3 (C-4´); 113.4 (CN); 127.3 (C-4´´); 128.9 (C-3´´ and C-5´´); 129.4 (C-2´´ and C-6´´) ; 131.5 (C-1´´); 145.7 (C-3´); 151.9 (C-5´); 163.0 (C-2); 164.3 (C-5). HRMS (ESI) calc. for C_12_H_8_N_6_SNa= 291.0429; found [M+Na]^+^ 291.0431.

5-amino-1-(5-(2-chlorophenyl)-1,3,4-thiadiazol-2-yl)-1*H*-pyrazole-4-carbonitrile **(9b)**. Yield: 55%. brown solid. MP: 250-251 ºC. IR (cm^-1^): 3388; 3295; 3232; 3190; 3143; 2924; 2359; 2223; 1628; 1576; 1506; 1479; 1431; 1407; 1368; 1294; 1228; 1193; 1162; 1127; 1064; 1039; 981; 921; 899; 873. ^1^H NMR (400 MHz, DMSO-d_6_, TMS, *δ* in ppm): 7.55-7.65 (m, 2H, H-4´and H-5´); 7.71-7.73 (m, 1H, H-3´´); 8.07 (s, 1H, H-3´); 8.13 (s, 2H, NH_2_); 8.17-8.20 (m, 1H, H-6´´). ^13^C NMR (100 MHz, DMSO-d_6_, TMS, *δ* in ppm): 73.4 (C-4´); 113.4 (CN); 127.6 (C-5´´); 127.9 (C-6´´); 130.7 (C-3´´); 131.4 (C-4´´); 132.6 (C-2´´); 145.9 (C-3´); 152.0 (C-5´); 159.6 (C-2); 164.2 (C-5). HRMS (ESI) calc. for C_12_H_7_ClN_6_SNa= 325.0039; found [M+Na]^+^ 325.0046.

5-amino-1-(5-(4-fluorophenyl)-1,3,4-thiadiazol-2-yl)-1*H*-pyrazole-4-carbonitrile **(9c)**. Yield: 91%. white solid. MP: 262-263 ºC. IR (cm^-1^): 3400; 3296; 3234; 3186; 3084; 2230; 1628; 1602; 1569; 1530; 1507; 1449; 1427; 1406; 1368; 1311; 1292; 1262; 1228; 1205; 1170; 1077; 984; 926; 882; 841; 812. ^1^H NMR (400 MHz, DMSO-d_6_, TMS, *δ* in ppm): 7.40-7.44 (m, 2H, H-3´´ and H-5´´); 8.04-8.08 (m, 2H, H-2´´ and H-6´´); 8.07 (s, 1H, H-3´); 8.11 (s, 2H, NH_2_). ^13^C NMR (100 MHz, DMSO-d_6_, TMS, *δ* in ppm): 73.4 (C-4´); 113.5 (CN); 116.6 (d, *J* = 22.3 Hz, C-3´´ and C-5´´); 125.6 (d, *J* = 3.2 Hz, C-2´´ and C-6´´); 129.9 (d, *J* = 9.0 Hz, C-1´´); 145.9 (C-3´), 152.0 (C-5´), 163.2 (C-2), 163.3 (C-5); 163.9 (d, *J* = 248 Hz, C-4´´). HRMS (ESI) calc. for C_12_H_7_FN_6_SNa= 309.0335; found [M+Na]^+^ 309.0344.

5-amino-1-(5-(4-chlorophenyl)-1,3,4-thiadiazol-2-yl)-1*H*-pyrazole-4-carbonitrile **(9d)**. Yield: 75%. white solid. MP: >300 ºC. IR (cm^-1^): 3402; 3295; 3234; 3186; 2920; 2233; 2030; 1979; 1630; 1596; 1575; 1530; 1510; 1444; 1428; 1399; 1384; 1367; 1297; 1265; 1214; 1165; 1091; 1075; 1024, 1014, 999; 983; 967; 931; 877; 835. ^1^H NMR (400 MHz, DMSO-d_6_, TMS, *δ* in ppm): 7.65 (d, 2H, *J* = 6.8 Hz, H-3´´ and H-5´´); 8.02 (d, 2H, J = 6.8 Hz, H-2´´ and H-6´´); 8.08 (s, 1H, H-3´); 8.12 (s, 2H, NH_2_). ^13^C NMR (100 MHz, DMSO-d_6_, TMS, *δ* in ppm): 73.2 (C-4´); 113.4 (CN); 127.8 (C-2´ and C-6´); 129.0 (C-3´´ and C-5´´); 129.5 (C-1´´); 136.2 (C-4´´); 145.9 (C-3´); 151.9 (C-5´); 163.2 (C-2); 163.3 (C-5). HRMS (ESI) calc. for C_12_H_7_ClN_6_SNa= 325.0039; found [M+Na]^+^ 325.0034

Ethyl 5-amino-1-(5-phenyl-1,3,4-thiadiazol-2-yl)-1*H*-pyrazole-4-carboxylate **(9e)**. Yield: 85%. brown solid. MP: 165-167 ºC. IR (cm^-1^): 3445; 3302; 3116; 3064; 2980; 2928; 2905; 2359; 2162; 1811; 1672; 1614; 155; 1539; 1519; 1501; 1462; 1428; 1398; 1376; 1353; 1315; 1285; 1264; 1205; 1157; 1111; 1074; 1030; 977; 920; 834. ^1^H NMR (400 MHz, DMSO-d_6_, TMS, *δ* in ppm): 1,28 (t, 3H, *J* = 7.1 Hz, CH_3_); 4.24 (q, 2H, *J* = 7.1 Hz, CH_2_); 7.46 (s, 2H, NH_2_); 7.55-7.60 (m, 3H, H-3´´, H-4´´ and H-5´´); 7.92 (s, 1H, H-3´); 7.98-8.00 (m, 2H, H-2´´ and H-6´´). ^13^C NMR (100 MHz, DMSO-d_6_, TMS, *δ* in ppm): 14.3 (CH_3_); 59.3 (CH_2_); 94.5 (C-4´); 127.3 (C-4´´); 128.9 (C-3´´ and C-5´´); 129.4 (C-2´´ and C-6´´); 131.5 (C-1´´); 144.3 (C-3´); 150.2 (C-5´); 162.5 (C-2); 163.3 (CO); 164.0 (C-5). HRMS (ESI) calc. for C_14_H_13_N_5_O_2_SNa= 3338.0688; found [M+Na]^+^ 338.0689.

Ethyl 5-amino-1-(5-(2-chlorophenyl)-1,3,4-thiadiazol-2-yl)-1*H*-pyrazole-4-carboxylate **(9f)**. Yield: 55%. brown solid. MP: 190-191 ºC. IR (cm^-1^): 3463; 3351; 3069; 2993; 2917; 2850; 2358; 2161; 2035; 1757; 1691; 1628; 1590; 1559; 1523; 1505; 1429; 1410; 1380; 1354; 1317; 1281; 1271; 1206; 1152; 1111; 1061; 1037; 979; 930; 879; 835; 810. ^1^H NMR (400 MHz, DMSO-d_6_, TMS, *δ* in ppm): 1.30 (t, 3H, *J* = 7.1Hz, CH_3_); 4.25 (q, 2H, *J* = 7.1 Hz, CH_2_); 7.50 (s, 2H, NH_2_); 7.56-7.66 (m, 2H, H-4´´ and H-5´´); 7.72-7.75 (m, 1H, H-2´´); 7.95 (s, 1H, H-3´); 8.18-8.20 (m, 1H, H-6´´). ^13^C NMR (100 MHz, DMSO-d_6_, TMS, *δ* in ppm): 14.3 (CH_3_); 59.4 (CH_2_); 94.5 (C-4´); 127.7 (C-5´´); 128.0 (C-6´´); 130.6 (C-3´´); 130.7 (C-4´´); 131.3 (C-2´´); 132.6 (C-1´´); 144.3 (C-3´); 150.3 (C-5´); 159.3 (C-2); 162.5 (CO); 164.5 (C-5). HRMS (ESI) calc. for C_14_H_12_ClN_5_O_2_SNa= 372.0298; found [M+Na]^+^  372.0292.

Ethyl 5-amino-1-(5-(4-fluorophenyl)-1,3,4-thiadiazol-2-yl)-1*H*-pyrazole-4-carboxylate **(9g)**. Yield: 77%. white solid. MP: 250-251 ºC. IR (cm^-1^): 3338; 3004; 2363; 1676; 1568; 1519; 1419; 1358; 1220; 1093; 903; 835. ^1^H NMR (400 MHz, DMSO-d_6_, TMS, *δ* in ppm): 1.29 (t, 3H, *J* = 7.1Hz, CH_3_); 4.25 (q, 2H, *J =* 7.1 Hz, CH_2_); 7.41-7.46 (m, 2H, H-3); 7.48 (s, 2H); 7.95 (s, 1H); 8.05-8.09 (m, 2H). ^13^C NMR (100 MHz, DMSO-d_6_, TMS, *δ* in ppm): 14.3 (CH_3_), 59.4 (CH_2_), 94.5 (C-4´), 116.6 (d, *J* = 22.3 Hz) (C-3´´ and C-5´´); 125.6 (C-1´´); 129.9 (d, *J* = 8.9 Hz) (C-2´´ and C-6´´); 144.3 (C-3´); 150.2 (C-5´); 162.5 (C-2); 162.9 (C-5); 163.4 (CO); 163.7 (d, *J* = 252.7 Hz) (C-4´´). HRMS (ESI) calc. for C_14_H_12_FN_5_O_2_SNa= 356.0594; found [M+Na]^+^  356.0604.

Ethyl 5-amino-1-(5-(4-chlorophenyl)-1,3,4-thiadiazol-2-yl)-1*H*-pyrazole-4-carboxylate **(9h)**. Yield: 77%. white solid. MP: 252-253 ºC. IR (cm^-1^): 3005; 2360; 2342; 1709; 1421; 1359; 1220; 1092; 902. ^1^H NMR (400 MHz, DMSO-d_6_, TMS, *δ* in ppm): 1.29 (t, 3H, *J* = 5.4 Hz, CH_3_); 4.25 (q, 2H, *J* = 5.4 Hz, CH_2_); 7.48 (s, 2H, NH_2_); 7.65-7.67 (m, 2H, H-3´´ and H-5´´); 7.96 (s, 1H, H-3´); 8.02-8.04 (m, 2H, H-2´´ and H-6´´). ^13^C NMR (100 MHz, DMSO-d_6_, TMS, *δ* in ppm): 14.3 (CH_3_); 59.4 (CH_2_); 94.5 (C-4´); 127.8 (C-3´´ and C-5´´); 129.0 (C-2´´ and C-6´´); 129.5 (C-1´´); 136.1 (C-4´´); 144.4 (C-3´); 150.2 (C-5´); 162.5 (C-2); 162.9 (CO); 163.6 (C-5). HRMS (ESI) calc. for C_14_H_12_ClN_5_O_2_SNa= 372.0298; found [M+Na]^+^  372.0292.

**1.2 General method for preparation of 2-(3,5-dimethyl-1*H*-pyrazol-1-yl)-1,3,4-thiadiazole (11a-d)**

The respective 2-hydrazinyl-1,3,4-thiadiazole (**7**) (2 mmol) and 2,4-pentanedione **(10)** (2.4 mmol) were dissolved in 40 mL of ethanol and the mixture was stirred and refluxed for 2-4 h. The reactions were accompanied by TLC (CHCl_3_/MeOH 9:1). After detecting the end of the reaction, the reaction medium was poured onto ice-cold water (50 mL). The product was precipitated, filtered, washed with cold water and recrystallized using the solvent mixture ethanol/water (3:1) to produce **11a-d** at 77- 95% yield.

2-(3,5-dimethyl-1*H*-pyrazol-1-yl)-5-phenyl-1,3,4-thiadiazole **(11a)**. Yield: 95%. brown solid. MP: 170-171 ºC. IR (cm^-1^): 3101; 2162; 1965; 1574; 1515; 1458; 1433; 1405; 1377; 1364; 1267; 1151; 1065; 1028; 998; 974; 910; 825. ^1^H NMR (400 MHz, DMSO-d_6_, TMS, *δ* in ppm): 2.22 (s, 3H, CH_3_); 2.67 (s, 3H, CH_3_); 6.29 (s, 1H, H-4´); 7.57-7.58 (m, 3H, H-3´´, H-4´´ and H-5´´); 7.97-7.99 (m, 2H, H-2´´ and H-6´´). ^13^C NMR (100 MHz, DMSO-d_6_, TMS, *δ* in ppm): 13.3 (CH_3_); 13.4 (CH_3_); 110.8 (C-4´); 127.4 (C-4´´); 129.5 (C-3´´ and C-5´´); 129.7 (C-2´´ and C-6´´); 131.6 (C-1´´); 142.4 (C-3´); 153.0 (C-5´); 163.6 (C-2); 164.7 (C-5). HRMS (ESI) calc. for C_13_H_12_N_4_SNa= 279.0681; found [M+Na]^+^ 279.0679.

2-(2-chlorophenyl)-5-(3,5-dimethyl-1*H*-pyrazol-1-yl)-1,3,4-thiadiazole **(11b)**. Yield: 82%. yellow solid. MP: 163-165 ºC. IR (cm^-1^): 3066; 2729; 2358; 1642; 1568; 1514; 1435; 1423; 1388; 1316; 1284; 1176; 1073; 1043; 994; 964; 884; 849. ^1^H NMR (400 MHz, DMSO-d_6_, TMS, *δ* in ppm): 2.23 (s, 3H, CH_3_); 2.69 (s, 3H, CH_3_); 6.30 (s, 1H, H-4´); 7.55-7.64 (m, 2H, H-4´´ and H-5´´); 7.72 (dd, 1H, *J* = 8.0 and 1.2 Hz, H-3´´); 8.16 (dd, 1H, *J* = 7.6 and 1.6 Hz, H-6´´). ^13^C NMR (100 MHz, DMSO-d_6_, TMS, *δ* in ppm): 13.2 (CH_3_); 13.2 (CH_3_); 110.7 (C-4´); 128.0 (C-5´´); 128.1 (C-6´´); 130.6 (C-3´´); 130.7 (C-4´´); 131.3 (C-2´´); 132.5 (C-1´´); 142.4 (C-3´); 153.0 (C-5´); 159.8 (C-2); 164.8 (C-5). HRMS (ESI) calc. for C_13_H_11_ClN_4_SNa= 313.0291; found [M+Na]^+^ 313.0283.

2-(3,5-dimethyl-1*H*-pyrazol-1-yl)-5-(4-fluorophenyl)-1,3,4-thiadiazole **(11c)**. Yield: 79%. white solid. MP: 174-174 ºC. IR (cm^-1^): 3081; 2919; 2850; 2359; 1596; 1577; 1558; 1515; 1450; 1406; 1385; 1297; 1266; 1218; 1165; 1134; 1105; 1075; 1024; 998; 966; 834; 810. ^1^H NMR (400 MHz, DMSO-d_6_, TMS, *δ* in ppm): 2.23 (s, 3H, CH_3_); 2.67 (s, 3H, CH_3_); 6.30 (s, 1H, H-4´); 7.41-7.45 (m, 2H, H-3´´ and H-5´´); 8.03-8.07 (m, 2H, H-2´´ and H-6´´). ^13^C NMR (100 MHz, DMSO-d_6_, TMS, *δ* in ppm): 13.1 (CH_3_); 13.1 (CH_3_); 110.5 (C-4´); 116.5 (d, *J* = 22.1 Hz) (C-3´´ and C-5´´); 126.0 (d, *J* = 3.3 Hz) (C-1´´); 129.6 (d, *J* = 8.8 Hz) (C-2´´ and C-6´´); 142.1 (C-3´); 152.7 (C-5´); 163.3 (C-2); 163.4 (C-5); 163.6 (d, *J* = 248.0 Hz) (C-4´´). HRMS (ESI) calc. for C_13_H_11_FN_4_SNa= 297.0586; found [M+Na]^+^ 297.0585.

2-(4-chlorophenyl)-5-(3,5-dimethyl-1*H*-pyrazol-1-yl)-1,3,4-thiadiazole **(11d)**. Yield: 77%. white solid. MP: 202-203 ºC. IR (cm^-1^): 3089; 2932; 2360; 2344; 2163; 1980; 1904; 1593; 1578; 1519; 1446; 1398; 1382; 1302; 1264; 1177; 1137; 1120; 1088; 1073; 1026; 985; 969; 826. ^1^H NMR (400 MHz, DMSO-d_6_, TMS, *δ* in ppm): 2.23 (s, 3H, CH_3_); 2.67 (s, 3H, CH_3_); 6.30 (s, 1H, H-4´); 7.64-7.66 (m, 2H, H-3´´ and H-5´´); 7.99-8.02 (m, 2H, H2´´ and H-6´´). ^13^C NMR (100 MHz, DMSO-d_6_, TMS, *δ* in ppm): 13.1 (CH_3_); 13.1(CH_3_); 110.6 (C-4´); 128.2 (C-2´ and C-6´); 128.8 (C-3´´ and C-5´´); 129.4 (C-1´´); 135.8 (C-4´´); 142.2 (C-3´); 152.8 (C-5´); 163.2 (C-2); 163.6 (C-5). HRMS (ESI) calc. for C_13_H_11_ClN_4_SNa= 313.0291; found [M+Na]^+^ 313.0286.

**1.3 General procedure for preparation of 5-methyl-1-(5-phenyl-1,3,4-thiadiazol-2-yl)-3-(trifluoromethyl)-4,5-dihydro-1*H*-pyrazol-5-ol (13a-d)**

A mixture of respective 2-hydrazinyl-5-phenyl-1,3,4-thiadiazoles **(7)** (2.0 mmol) and 1,1,1-trifluoropentane-2,4-dione **(10)** (2.4 mmol) and 40 mL of ethanol was kept under stirring and reflux for 2-6 h. The advance of the reaction was monitored using TLC (CHCl_3_/MeOH 9:1). After detecting the end of the reaction, the reaction medium was poured onto ice-cold water (50 mL). The precipitate produced was selected by filtration, washed with water and recrystallized from ethanol/water (3:1) to produce **13a-d** at 54- 90% yield.

5-methyl-1-(5-phenyl-1,3,4-thiadiazol-2-yl)-3-(trifluoromethyl)-4,5-dihydro-1*H*-pyrazol-5-ol **(13a)**. Yield: 79%. yellow solid. MP: 183-185 ºC. IR (cm^-1^): 3005; 2364; 2338; 1713; 1646; 1577; 1521; 1465; 1438; 1363; 1310; 1280; 1222; 1197; 1173; 1159; 1078; 995. ^1^H NMR (400 MHz, DMSO-d_6_, TMS, *δ* in ppm): 2.07 (s, 3H, CH_3_); 3.23 (d, 1H, *J* = 19.2 Hz, H_a_4´ or H_b_4´); 3.63 (d, 1H, *J* = 19.2 Hz, H_a_4´ or H_b_4´); 7.50-7.53 (m, 3H, H-3´´, H-4´´ and H-5´´); 7.86-7.88 (m, 2H, H-2´´ and H-6´´); 8.31 (s, 1H, OH). ^13^C NMR (100 MHz, DMSO-d_6_, TMS, *δ* in ppm): 15.5 (CH_3_); 48.5 (C-4´); 92.3 (q, *J* = 33.7 Hz) (C-3´); 123.2 (q, *J* = 271.6 Hz) (CF_3_); 126.8 (C-4´´); 129.3 (C-3´´ and C-5´´); 130.2 (C-2´´ and C-6´´); 130.5 (C-1´´); 155.9 (C-5´); 161.1 (C-2); 164.9 (C-5). HRMS (ESI) calc. for C_13_H_11_F_3_N_4_OSNa= 351.0504; found [M+Na]^+^ 351.0500.

1-(5-(2-chlorophenyl)-1,3,4-thiadiazol-2-yl)-5-methyl-3-(trifluoromethyl)-4,5-dihydro-1*H*-pyrazol-5-ol **(13b)**. Yield: 54%. white solid. MP: 163-165 ºC. IR (cm^-1^): 3065; 2728; 2358; 1642; 1567; 1514; 1435; 1432; 1387; 1316; 1283; 1175; 1073; 1092; 994; 964; 887; 850. ^1^H NMR (400 MHz, DMSO-d_6_, TMS, *δ* in ppm): 2.08 (s, 3H, CH_3_); 3.24 (d, 1H, *J* = 19,2 Hz, H_a_4´ or H_b_4´); 3.64 (d, 1H, *J* = 19.2 Hz, H_a_4´ or H_b_4´); 7.52-7.57 (m, 2H, H-4´´ and H-5´´); 7.65-7.67 (m, 1H, H-3´´); 8.07-8.10 (m, 1H, H-6´´); 8.35 (s, 1H, OH). ^13^C NMR (100 MHz, DMSO-d_6_, TMS, *δ* in ppm): 15.2 (CH_3_); 48.5 (C-4´); 92.3 (q, *J* = 33.0 Hz, C-3´); 123.1 (q, *J* = 281.1 Hz, CF_3_); 127.8 (C-5´´); 128.9 (C-6´´); 130.5 (C-3´´); 130.5 (C-4´´); 131.0 (C-2´´); 131.7 (C-1´´); 155.9 (C-5´); 156.2 (C-2); 166.1 (C-5). HRMS (ESI) calc. for C_13_H_10_ClF_3_N_4_OSH= 363.0284; found [M+H]^+^ 363.0295.

1-(5-(4-fluorophenyl)-1,3,4-thiadiazol-2-yl)-5-methyl-3-(trifluoromethyl)-4,5-dihydro-1*H*-pyrazol-5-ol **(13c)**. Yield: 90%. white solid. MP: 182-183 ºC. IR (cm^-1^): 3077; 1713; 1601; 1592; 1526; 1467; 1388; 1362; 1311; 1279; 1266; 1240; 1202; 1171; 1162; 1154; 1129; 1099; 1074; 1046; 985; 841; 816. ^1^H NMR (400 MHz, DMSO-d_6_, TMS, *δ* in ppm): 2.07 (s, 3H, CH_3_); 3.23 (d, 1H, *J* = 19.2 Hz, H_a_4´ or H_b_4´ ); 3.63 (d, *1H*, *J* = 19.2 Hz, H_a_4´ or H_b_4´); 7.35-7.38 (m, 2H, H-3´´ and H-5´´); 7.92-7.95 (m, 2H, H-2´´ and H-6´´); 8.33 (s, 1H, OH). ^13^C NMR (100 MHz, DMSO-d_6_, TMS, *δ* in ppm): 15.1 (CH_3_); 48.4 (C-4´); 92.2 (q, *J* = 33.1 Hz, C-3´); 116.3 (d, *J* = 22.0 Hz, C-3´´ and C-5´´); 123.0 (q, *J* = 283.3 Hz, CF_3_); 126.7 (d, *J* = 3.0 Hz, C-1´´); 129.0 (d, *J* = 8.7 Hz, C-2´´ and C-6´´); 155.7 (C-5´); 159.7 (C-2); 163.3 (d, *J =* 274.0 Hz, C-4´´); 164.4 (C-5). HRMS (ESI) calc. for C_13_H_10_F_4_N_4_OSNa= 369.0409; found [M+Na]^+^ 369.0410.

1-(5-(4-chlorophenyl)-1,3,4-thiadiazol-2-yl)-5-methyl-3-(trifluoromethyl)-4,5-dihydro-1*H*-pyrazol-5-ol **(13d)**. Yield: 62%. white solid. MP: 184-185 ºC. IR (cm^-1^): 3056; 2360; 1640; 1595; 1569; 1526; 1459; 1387; 1349; 1313; 1274; 1197; 1179; 1173; 1160; 11301; 1105; 1095; 1073; 1015; 994; 983; 960; 882; 839; 822. ^1^H NMR (400 MHz, DMSO-d_6_, TMS, *δ* in ppm): 2.08 (s, 3H, CH_3_); 3,24 (d, 1H, *J* = 19.2 Hz, H_a_4´ or H_b_4´); 3.65 (d, 1H, *J* = 19.2 Hz, H_a_4´ or H_b_4´); 7.57-7.60 (m, 2H, H-3´´ and H-5´´); 7.88-7.92 (m, 2H, H-2´´ and H-6´´); 8.36 (s, 1H, OH). ^13^C NMR (100 MHz, DMSO-d_6_, TMS, *δ* in ppm): 15.1 (CH_3_); 48.3 (C-4´); 92.2 (q, *J* = 33.2 Hz, C-3´); 123.0 (q, *J* = 283.4 Hz, CF_3_); 128.3 (C-2´´ and C-6´´); 129.0 (C-3´´ and C-5´´); 129.3 (C-1´´); 134.9 (C-4´´); 155.8 (C-5´); 159.6 (C-2); 164.8 (C-5). HRMS (ESI) calc. for C_13_H_10_ClF_3_N_4_OSNa= 385.0114; found [M+Na]^+^ 385.0106.

**1.4 General procedure for preparation of 2-(5-methyl-3-(trifluoromethyl)-1*H*-pyrazol-1-yl)-1,3,4-thiadiazole (14a-d)**

The respective 5-methyl-1-(5-phenyl-1,3,4-thiadiazol-2-yl)-3-(trifluoromethyl)-4,5-dihydro-1*H*-pyrazol-5-ol (**13a-d**) (1.0 mmol) was dissolved in 5 mL of pyridine and the reaction was maintained at 0 ^o^C and 1.5 mL of thionyl chloride was added slowly. The reaction mixture was stirred in room temperature for 12 h. The reactions were accompanied by TLC (CHCl_3_/MeOH 9:1). After detecting the end of the reaction, was added 22 mL of HCl 3 M, washed with water (30 mL) and extracted with chloroform (3 × 30 mL). The organic phase was dried (magnesium sulfate), filtered, and evaporated under vacuum. Compounds **14a-d** were obtained in 60-96% yield.

2-(5-methyl-3-(trifluoromethyl)-1*H*-pyrazol-1-yl)-5-phenyl-1,3,4-thiadiazole **(14a)**. Yield: 96%. yellow solid. MP: 163-164 ºC. IR (cm^-1^): 3119; 2936; 2163; 1980; 1600; 1579; 1522; 1460; 1435; 1388; 1298; 1189; 1141; 1064; 1021; 951; 921; 839; 762; 688; 621; 587; 537. ^1^H NMR (400 MHz, DMSO-d_6_, TMS, *δ* in ppm): 2.35 (s, 3H, CH_3_); 7.24 (s, 1H, H-4´); 7.55-7.61 (m, 3H, H-3´´, H-4´´ and H-5´´); 7.99-8.02 (m, 2H, H-2´´ and H-6´´). ^13^C NMR (100 MHz, DMSO-d_6_, TMS, *δ* in ppm): 13.4 (CH_3_); 112.7 (q, *J* = 2.7 Hz, C-4´); 119.1 (q, *J* = 267.9 Hz, CF_3_); 127.6 (C-4´´); 129.2 (C-3´´ and C-5´´); 129.7 (C-2´´ and C-6´´); 131.5 (C-1´´); 133.4 (q, *J* = 41.7 Hz, C-3´); 152.3 (C-5´); 161.0 (C-2), 167.0 (C-5). HRMS (ESI) calc. for C_13_H_9_F_3_N_4_SNa= 333.0398; found [M+Na]^+^ 333.0408.

2-(2-chlorophenyl)-5-(5-methyl-3-(trifluoromethyl)-1*H*-pyrazol-1-yl)-1,3,4-thiadiazole **(14b)**. Yield: 60%. white solid. MP: 139-140 ºC. IR (cm^-1^): 3170; 3122; 2925; 2856; 2163; 1984; 1942; 1916; 1830; 1747; 1653; 1586; 1519; 1474; 1389; 1367; 1230; 1221; 1134; 1069; 982; 956; 841; 761; 729; 662; 526. ^1^H NMR (400 MHz, DMSO-d_6_, TMS, *δ* in ppm): 2.40 (s, 3H, CH_3_); 6.75 (s, 1H, H-4´); 7.42-7.48 (m, 2H, H-4´´ and H-5´´); 7.53-7.56 (m, 1H, H-3´´); 8.41-8.43 (m, 1H, H-6´´). ^13^C NMR (100 MHz, DMSO-d_6_, TMS, *δ* in ppm): 13.5; 112.7-112.8 (m, C-4´); 119.1 (q, *J* = 267.7 Hz, CF_3_); 127.5 (C-5´´); 128.6 (C-6´´); 130.6 (C-3´´); 130.7 (C-4´´); 131.9 (C-2´´); 132.4 (C-1´´); 133.5 (q, *J =* 41.7 Hz, C-3´); 152.4 (C-5´); 162.2 (C-2); 162.7 (C-5). HRMS (ESI) calc. for C_13_H_8_ClF_3_N_4_SNa= 367.0008; found [M+Na]^+^ 366.9992.

2-(4-fluorophenyl)-5-(5-methyl-3-(trifluoromethyl)-1*H*-pyrazol-1-yl)-1,3,4-thiadiazole **(14c)**. Yield: 79%. white solid. MP: 149-150 ºC. IR (cm^-1^): 3167; 3085; 2161; 2034; 1752; 1670; 1597; 1577; 1514; 1447; 1390; 1369; 1291; 1224; 1135; 1066; 983; 955; 817; 762; 667; 620; 584; 527. ^1^H NMR (400 MHz, DMSO-d_6_, TMS, *δ* in ppm): 2.35 (s, 3H, CH_3_); 7.26 (s, 1H, H-4´); 7.41-7.47 (m, 2H, H-3´´ and H-5´´); 8.06-8.11 (m, 2H). ^13^C NMR (100 MHz, DMSO-d_6_, TMS, *δ* in ppm): 13.0; 113.9-114.0 (m, C-4´); 116.7 (d, *J* = 22.3 Hz) (C-3´´ and C-5´´); 119.1 (q, *J* = 267.4 Hz, CF_3_); 125.7 (d, *J* = 3.0 Hz, C-1´´); 130.0 (d, *J* = 8.9 Hz, C-2´´ and C-6´´); 131.6 (q, *J* = 41.0 Hz, C-3´); 152.9 (C-5´); 161.0 (C-2); 164.0 (d, *J* = 248.7 Hz, C-4´´); 165.5 (C-5) (d, *J* = 21.7 Hz). HRMS (ESI) calc. for C_13_H_8_F_4_N_4_SNa= 351.0304; found [M+Na]^+^ 351.0298.

2-(4-chlorophenyl)-5-(5-methyl-3-(trifluoromethyl)-1*H*-pyrazol-1-yl)-1,3,4-thiadiazole **(14d)**. Yield: 70%. brown solid. MP: 147-148 ºC. IR (cm^-1^): 3156; 3081; 2162; 1963; 1594; 1576; 1525; 1445; 1390; 1368; 1294; 1219; 1182; 1140; 1090; 1066; 981; 953; 852; 818; 777; 732; 656; 581; 538. ^1^H NMR (400 MHz, DMSO-d_6_, TMS, *δ* in ppm): 2.35 (s, 3H, CH_3_); 7.26 (s, 1H, H-4´); 7.64-7.67 (m, 2H, H-3´´ and H-5´´); 8.02-8.05 (m, 2H, H-2´´ and H-6´´). ^13^C NMR (100 MHz, DMSO-d_6_, TMS, *δ* in ppm): 13.0 (CH_3_); 114.0; 119.1 (q, *J* = 267.4 Hz) (CF_3_); 127.9 (C-2´´ and C-6´´); 129.*2* (C-3´´ and C-5´´); 129.6 (C-1´´); 131.6 (q, *J* = 41.4 Hz) (C-3´); 136.4 (C-4´´); 152.9 (C-5´); 161.2 (C-2); 165.4 (C-5). HRMS (ESI) calc. for C_13_H_8_ClF_3_N_4_SNa= 367.0008; found [M+Na]^+^ 367,0003.
