# Supplementary figures and images for: Synthesis, Biological Evaluation, and Molecular Modeling Studies of New Thiadiazole Derivatives as Potent P2X7 Receptor Inhibitors
Source: Front Chem. 2019 Apr 30;7:261. doi: 10.3389/fchem.2019.00261 (PMC6511888; doi:10.3389/fchem.2019.00261)

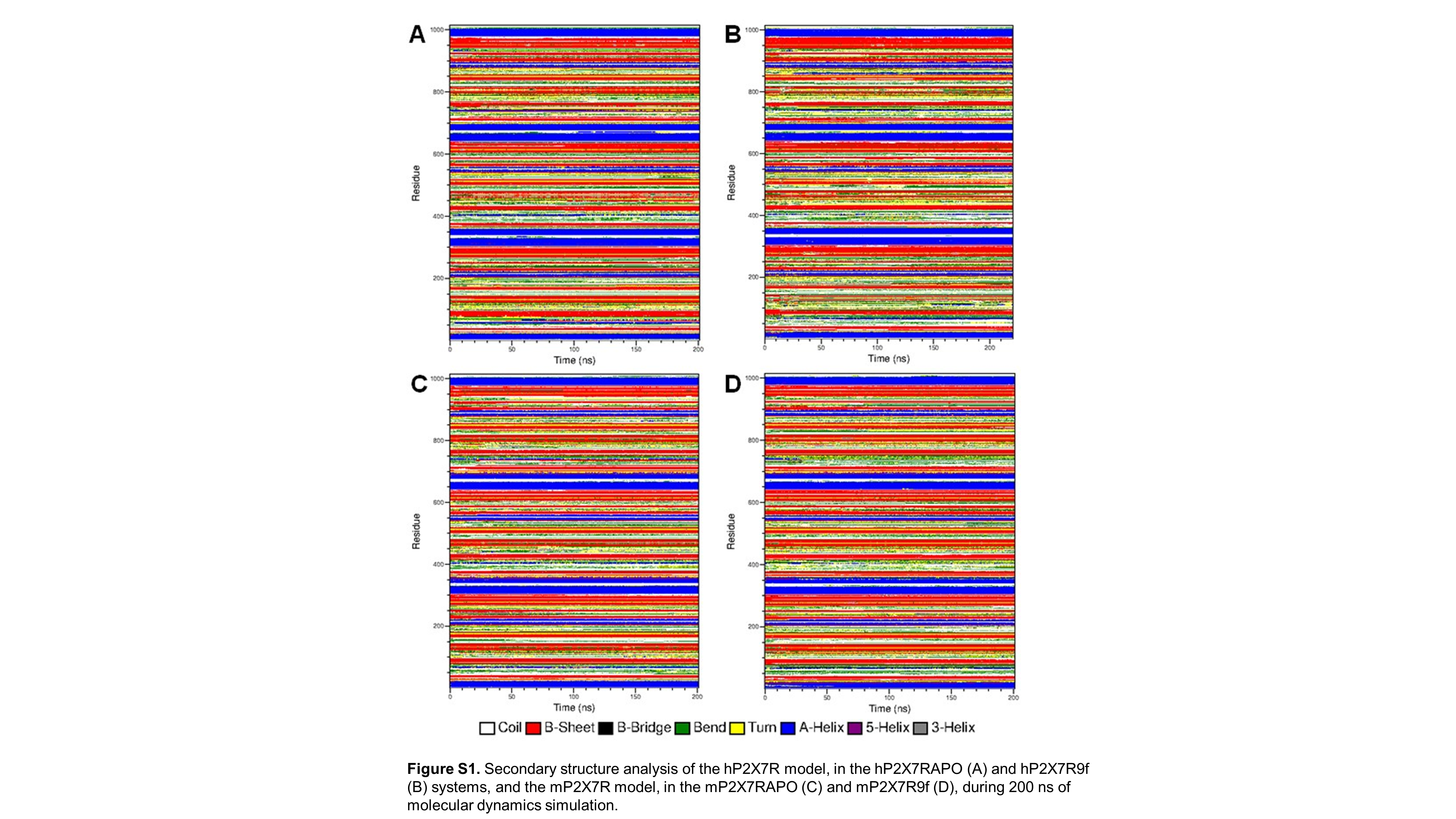

Supplement: Supplementary file 4 [file Image_1.tif]
